# Colorectal cancer cells utilize autophagy to maintain mitochondrial metabolism for cell proliferation under nutrient stress

**DOI:** 10.1172/jci.insight.138835

**Published:** 2021-06-17

**Authors:** Samantha N. Devenport, Rashi Singhal, Megan D. Radyk, Joseph G. Taranto, Samuel A. Kerk, Brandon Chen, Joshua W. Goyert, Chesta Jain, Nupur K. Das, Katherine Oravecz-Wilson, Li Zhang, Joel K. Greenson, Y. Eugene Chen, Scott A. Soleimanpour, Pavan Reddy, Costas A. Lyssiotis, Yatrik M. Shah

**Affiliations:** 1 Cellular and Molecular Biology,; 2 Departments of Molecular & Integrative Physiology,; 3 Hematology & Oncology,; 4 Department of Pathology,; 5 Cardiovascular Center,; 6 Metabolism, Endocrinology & Diabetes,; 7 Rogel Cancer Center, and; 8 Internal Medicine, Division of Gastroenterology, University of Michigan Medical School, Ann Arbor Michigan, USA.

**Keywords:** Gastroenterology, Oncology, Colorectal cancer

## Abstract

Cancer cells reprogram cellular metabolism to maintain adequate nutrient pools to sustain proliferation. Moreover, autophagy is a regulated mechanism to break down dysfunctional cellular components and recycle cellular nutrients. However, the requirement for autophagy and the integration in cancer cell metabolism is not clear in colon cancer. Here, we show a cell-autonomous dependency of autophagy for cell growth in colorectal cancer. Loss of epithelial autophagy inhibits tumor growth in both sporadic and colitis-associated cancer models. Genetic and pharmacological inhibition of autophagy inhibits cell growth in colon cancer–derived cell lines and patient-derived enteroid models. Importantly, normal colon epithelium and patient-derived normal enteroid growth were not decreased following autophagy inhibition. To couple the role of autophagy to cellular metabolism, a cell culture screen in conjunction with metabolomic analysis was performed. We identified a critical role of autophagy to maintain mitochondrial metabolites for growth. Loss of mitochondrial recycling through inhibition of mitophagy hinders colon cancer cell growth. These findings have revealed a cell-autonomous role of autophagy that plays a critical role in regulating nutrient pools in vivo and in cell models, and it provides therapeutic targets for colon cancer.

## Introduction

Autophagy is an important process involved in maintaining cellular homeostasis. Autophagy removes defective organelles and proteins through lysosomal breakdown. This process can occur via macroautophagy (herein referred to as autophagy), through the nonselective engulfment of cytoplasmic contents, or through selective autophagy, which targets specific cargo. In colon cancer, autophagy is found to have both pro- and antitumor functions in cancer-derived cell lines (1–3). Consistent with these data, studies have also found both beneficial and deleterious roles of autophagy in clinical outcomes in colorectal cancer (CRC) patients (4–6). Therefore, the function of autophagy in CRC remains unclear. In mouse models of CRC, intestinal epithelial disruption of *Atg7*, a gene involved in formation of the autophagosome membrane, led to decreased tumors (7). The work demonstrated that intestinal epithelial inhibition of autophagy promoted an antitumor immune response via alterations in the commensal microbiota population. These data are consistent with changes in the basal gut microbiota following intestinal epithelial *Atg5,* a protein involved in autophagic vesicle formation and disruption (8). The tumor microenvironment increases cell stress caused by decreasing oxygen availability and reducing nutrient supply. This cell stress is further exacerbated by the antitumor response. To adapt to limited oxygen and nutrients, cancer cells modify metabolic pathways to maintain growth. Autophagic products can replenish nutrient pools in cancer (9–15). However, this work has been done in KRAS mutant tumors, and very little is known with respects to contribution and integration of cellular autophagy to colon cancer cell metabolism and growth (9–15).

In the current study, we identified a cell-autonomous dependency of autophagy in colon cancer cell lines, patient-derived enteroids, and mouse models. Loss of epithelial autophagy in murine tumor models reduced overall tumor number, tumor burden, and proliferation. Consistent with these data, tumor growth and proliferation were significantly decreased in CRC patient–derived enteroid models, but not in normal enteroids. In nutrient-starved environments, colon cancer cells require autophagy to maintain cellular nutrient pools. Through metabolomics and lysosomal proteomics, mitophagy was rapidly initiated in low nutrient conditions, and recycling of mitochondrial metabolites was observed. Temporal knockdown of mitophagy led to decreased colon cancer cell growth in nutrient-rich cell culture conditions. These data demonstrate that CRCs are addicted to mitophagy to maintain cell growth. There are several clinical trials targeting autophagy for cancer treatment, and this work establishes a critical role of mitophagy in CRC growth (16).

## Results

### Intestinal epithelial disruption of autophagy inhibits colon tumor growth.

**Figure 1:**
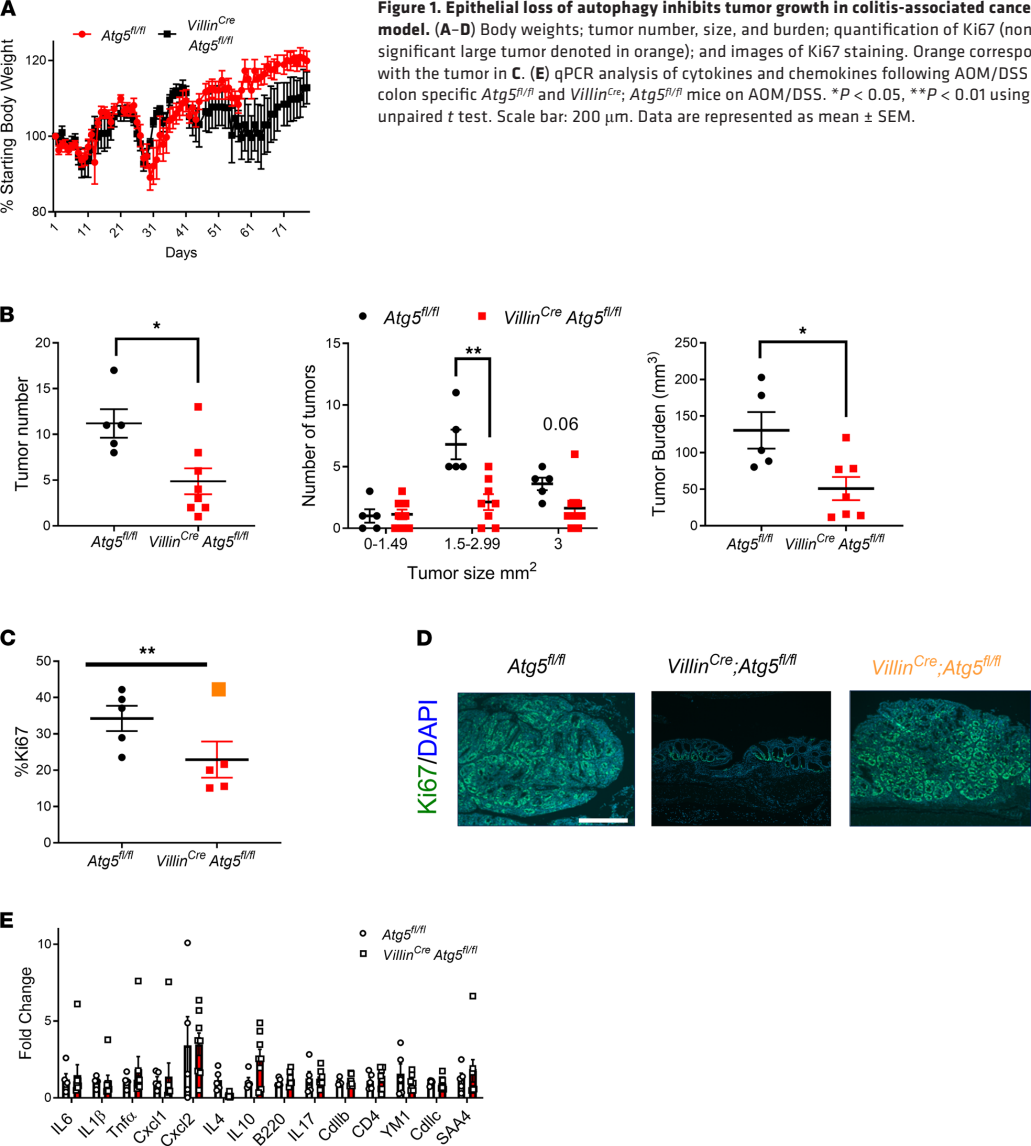
Epithelial loss of autophagy inhibits tumor growth in colitis-associated cancer model. (**A**–**D**) Body weights; tumor number, size, and burden; quantification of Ki67 (nonsignificant large tumor denoted in orange); and images of Ki67 staining. Orange corresponds with the tumor in **C**. (**E**) qPCR analysis of cytokines and chemokines following AOM/DSS in colon specific *Atg5^fl/fl^* and *Villin^Cre^*; *Atg5^fl/fl^* mice on AOM/DSS. *P < 0.05, **P < 0.01 using unpaired *t* test. Scale bar: 200 μm. Data are represented as mean ± SEM.

**Figure 2:**
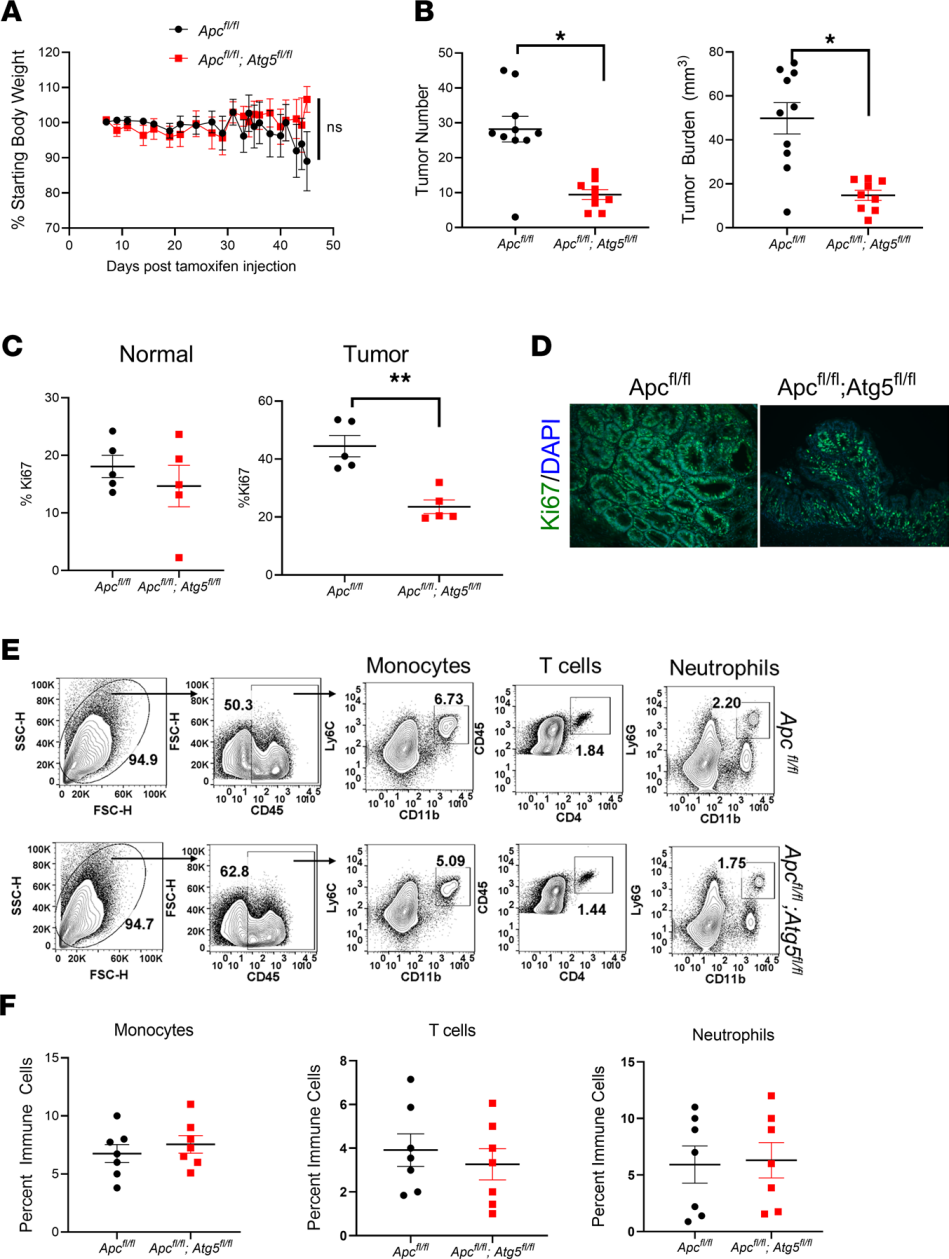
Epithelial loss of autophagy inhibits tumor growth in a sporadic colon cancer model. (**A**–**D**) Body weights, tumor number and burden, quantification of Ki67 staining from normal and tumor tissue, and a representative image of Ki67 staining. (**E**) Representative flow blot demonstrating gating strategy of immune cell types. (**F**) Quantitation from flow cytometry of immune cells in *Cdx2-ER^T2^*Cre;*Apc^fl/fl^* (*n* = 10; tumor quantitation and *n* = 7; flow cytometry) and *Cdx2-ER^T2^*Cre;*Apc^fl/fl^*;*Atg5^fl/fl^* (*n* = 9; tumor quantitation and *n* = 7; flow cytometry) mice. Tumors were assessed at 6 weeks following tamoxifen treatment, and flow cytometry was assessed at 2 weeks following tamoxifen treatment. *P < 0.05, **P < 0.01 using unpaired *t* test. Data are represented as mean ± SEM. This experiment was repeated twice.

*Atg5^fl/fl^* mice were crossed to mice expressing Cre recombinase from the Villin promoter to specifically target intestinal epithelial cells (Supplemental Figure 1, A–C; supplemental material available online with this article; https://doi.org/10.1172/jci.insight.138835DS1). The azoxymethane (AOM) and dextran sulfate sodium (DSS) model is an established colitis-associated cancer (CAC) model that specifically develops colon tumors. In the AOM/DSS model, *Atg5^fl/fl^* and *Villin^Cre^*;*Atg5^fl/fl^* mice showed no significant difference in body weight, although a slight decrease was noted in the *Villin^Cre^*;*Atg5^fl/fl^* mice during the final cycle of DSS (Figure 1A). The *Villin^Cre^*;*Atg5^fl/fl^* mice demonstrated a decrease in tumor number and burden (Figure 1B). Tumors from *Villin^Cre^*;*Atg5^fl/fl^* mice had reduced proliferation as measured by Ki67 staining (Figure 1, C and D). However, we did observe — in the few rare large tumors from the *Villin^Cre^*;*Atg5^fl/fl^* — that proliferation was comparable with *Atg5^fl/fl^* mice (Figure 1, C and D). Previous work investigating loss of *Atg7* in intestinal epithelial cells highlighted the impact of the immune response and gut microbiota in tumors (7). Cytokine and chemokine mRNA were measured from the colon tissue, and no change was found between the *Atg5^fl/fl^* or *Villin^Cre^*;*Atg5^fl/fl^* mice (Figure 1E). Similarly, loss of intestinal epithelial autophagy did not alter disease susceptibility to acute colitis induced by DSS. No changes in weight, colon length, or inflammation score as determined by a blinded pathologist were noted (Supplemental Figure 1, D–G). Expression of cytokines and chemokines was not altered with loss of autophagy (Supplemental Figure 1H). Transcription factor EB (TFEB) activates genes involved in autophagosome formation, cargo recognition, and fusion with the lysosome. When autophagy was disrupted by loss of TFEB in a tamoxifen-inducible *Vil-ER^T2^*;*Tfeb^fl/fl^* model, there was no change in weight, colon length, or inflammation score (Supplemental Figure 2, A–F). However, proinflammatory mediators were significantly increased (Supplemental Figure 2G). While we did not observe effects of autophagy loss on the response to acute colitis, others have clearly demonstrated a role for intestinal epithelial autophagy in colitis severity (17–19). Genome-wide association studies have linked polymorphisms of many known autophagic genes to susceptibility for ulcerative colitis or Crohn’s disease (20, 21). Moreover, the data from the *Vil-ER^T2^Cre*;*Tfeb^fl/fl^* model show increased proinflammatory mediators following injury. Therefore, the development of tumors through AOM/DSS is confounded by inflammation-driven tumor development. A sporadic colon tumor model was generated by crossing the known, adenomatous polyposis coli model, *Apc^fl/fl^* mice*,* or double *Apc^fl/fl^*;*Atg5^fl/fl^* mice to a tamoxifen-inducible colon–specific *Cdx2-ER^T2^Cre* (22). Mice were induced with a single dose (50 mg/kg) of tamoxifen, and 6 weeks following injections, tissues were collected. Mice showed no difference in body weight (Figure 2A). The *Cdx2-ER^T2^Cre*;*Apc^fl/fl^*;*Atg5^fl/fl^* showed a significant reduction in tumor number and burden compared with *Cdx2-ER^T2^Cre*;*Apc^fl/fl^* mice (Figure 2B). Proliferation measured by Ki67 was reduced with loss of *Atg5* (Figure 2, C and D). Adjacent normal tissue showed no difference in proliferation with autophagy loss (Figure 2C). To investigate if infiltration of immune cells was altered in the sporadic model following loss of autophagy, flow cytometry analysis of abundant immune populations was assessed. Two weeks following tamoxifen induction, immune cells were isolated from the colon. No difference was observed between the relative monocyte, T cell, or neutrophil populations (Figure 2, E and F). In inflammatory bowel disease (IBD), Paneth cells are particularly impacted by changes in autophagy (23, 24). Therefore, we performed gene expression analysis of Paneth cell markers in our *Cdx2-ER^T2^Cre*;*Apc^fl/fl^*;*Atg5^fl/fl^* cohort and found no changes in Paneth cells with loss of autophagy in either tumor or matched normal tissue (Supplemental Figure 3).

### Autophagy loss inhibits tumor proliferation in a cell-autonomous manner.

**Figure 3:**
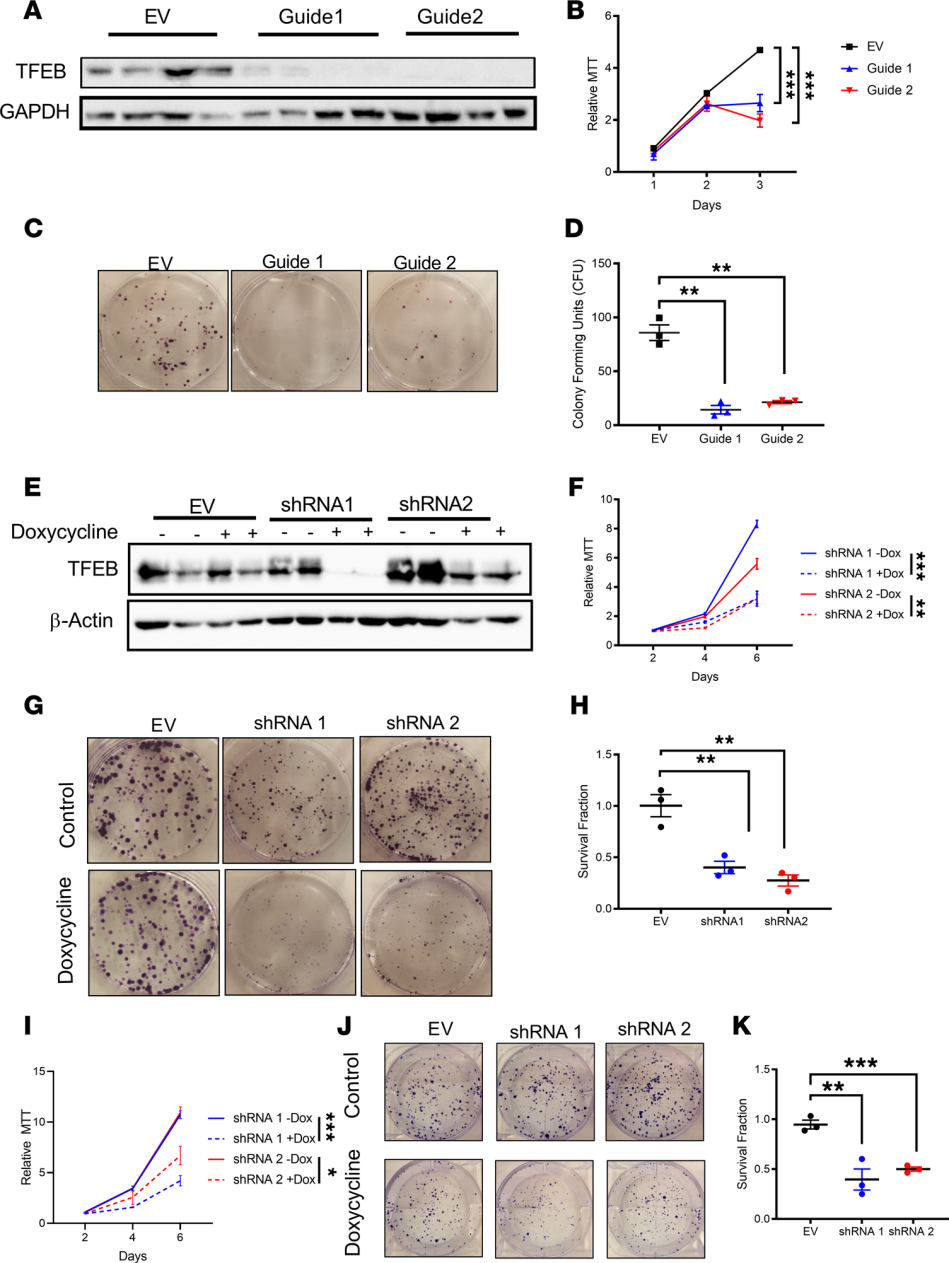
Cell-autonomous inhibition of autophagy inhibits cell growth. (**A**–**D**) Western blot analysis, MTT assay, representative images of clonogenic assay, and quantification by blinded observers in stable HCT116 expressing empty vector (EV) or 2 different gRNAs specific for *TFEB* (Guide 1 and Guide 2). (**E**–**H**) Western blot analysis, MTT assay, representative images of clonogenic assay, and quantification of clonogenics by blinded observers in doxycycline-inducible shRNA specific for TFEB (shRNA 1 and shRNA 2) or EV in HCT116. (**I**–**K**) MTT assay, representative images of clonogenic assay, and quantification of clonogenics by blinded observers in doxycycline-inducible shRNA specific for TFEB (shRNA 1 and shRNA 2) or EV in SW480. *P < 0.05, **P < 0.01, ***P < 0.001 using 2-way ANOVA with Tukey’s multiple-comparison test. Data are represented as mean ± SEM. All experiments were done in triplicates and repeated 3 times.

**Figure 4:**
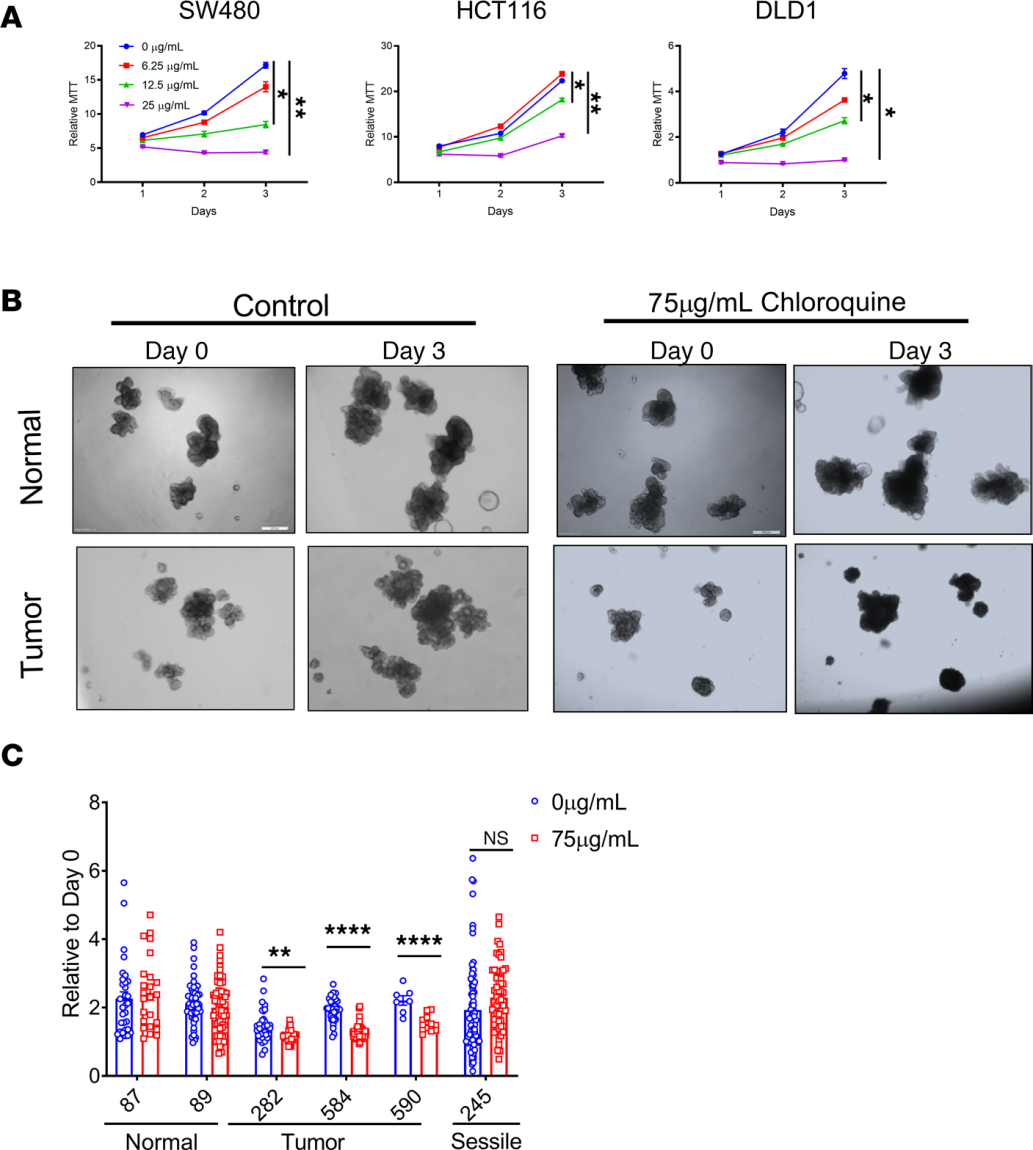
Pharmacological inhibition of autophagy inhibits CRC growth. (**A**) MTT assay in colon cancer–derived cell lines (SW480, HCT116, and DLD1) with chloroquine treatment. (**B** and **C**) Representative images and growth quantification of normal and colon cancer patient–derived enteroids treated with chloroquine for 3 days. Scale bar: 500 μm *P < 0.05, **P < 0.01, ****P < 0.0001 using unpaired *t* test. Data are represented as mean ± SEM. The CRC cell line experiments were done in triplicate and repeated 3 times. The enteroid work was done once in triplicate with the indicated lines.

The lack of inflammatory changes in parallel with reduced tumor burden led us to investigate the cell autonomous role of autophagy. CRISPR/Cas9-mediated disruption of TFEB in CRC-derived HCT116 cells (Figure 3A) showed a marked reduction in growth as assessed by MTT and long-term clonogenic cell survival assays (Figure 3, B–D). In addition, doxycycline-inducible shRNAs for TFEB in HCT116 and SW480 cell lines demonstrated reduced growth, whereas doxycycline-treated empty vector cells were not changed (Figure 3, E–K, and Supplemental Figure 4A). ATG4B is an essential regulator of autophagy (25). Stable HCT116 cells expressing a dominant-negative *ATG4B^C74A^* mutant demonstrated decreased growth by MTT and clonogenic analysis (Supplemental Figure 4, B and C) (26). Pharmacological inhibition of autophagy is currently in clinical trials for a number of cancers (NCT02333890, NCT02378532, and NCT03400865; https://clinicaltrials.gov) To understand the impact of pharmacological inhibition, growth in CRC-derived cell lines was measured following treatment with chloroquine, a lysosomal inhibitor. In CRC-derived cell lines (SW480, HCT116, and DLD1), increasing doses of chloroquine led to a marked reduction in cell growth (Figure 4A). A similar response was observed in CRC-derived HT29 and RKO cell lines and in mouse MC38, and CT26 cell lines (Supplemental Figure 5A). Autophagy can be activated by serine/threonine protein kinase 1 (ULK1) (27). Inhibition of ULK1 with SBI-0206965 also reduced cell growth similarly to chloroquine (Supplemental Figure 5B). Cell growth was rescued when low-dose (but not high-dose) chloroquine was removed (Supplemental Figure 5C).

To assess if the impact of autophagy loss to cell growth was selective to tumor cells, 4 patient-derived tumor enteroids and 2 normal colon enteroids were assessed (28). Enteroids 282, 584, and 590 are adenomas located in the ascending colon, and enteroid 245 is an adenoma that is sessile serrated from the cecum. Patient-derived tumor enteroids demonstrated significant growth inhibition at day 3 of chloroquine treatment when compared with day 0. However, similar growth inhibition was not observed in normal colon enteroids, which did not demonstrate any growth defects following inhibition of autophagy (Figure 4, B and C, and Supplemental Figure 5D). It is interesting to note that a sessile serrated tumor enteroid did not respond to autophagy inhibition. Sessile serrated tumors are a recently recognized class of colon cancers that present with *BRAF* mutations, as opposed to *APC* mutations, which are seen in the majority of colon cancer (29, 30). The inhibition of growth highlights a dependency on autophagy in tumor cells that is not observed in normal tissue.

### Tumor cells rely on autophagy under states of limited nutrient availability.

**Figure 5:**
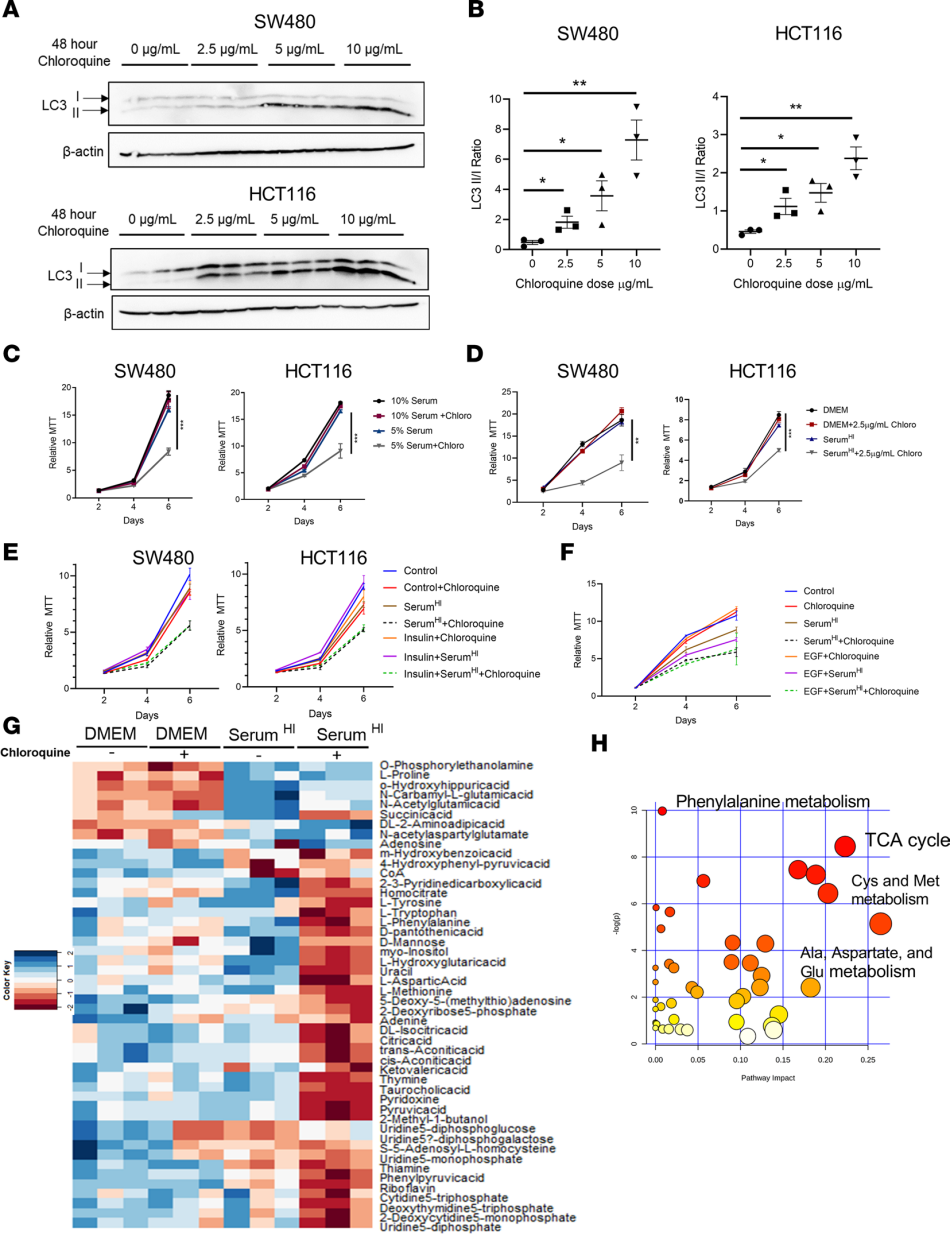
Nutrient stress requires autophagy to maintain cell growth. (**A** and **B**) Western blot and quantification of chloroquine dose to inhibit autophagy in HCT116 and SW480 determined by the LC3I/II ratio. (**C**) MTT assay; cells were cultured in DMEM with 5% or 10% serum and in combination with chloroquine at 2.5 μg/mL. (**D**) MTT assay; cells were cultured in DMEM with normal or Serum^hi^ in combination with chloroquine at 2.5 μg/mL. (**E** and **F**) Supplementation of cells treated with Serum^hi^ and chloroquine with insulin (10 nM) in HCT116 and SW480 or hEGF (50nM) in HCT116. **E** and **F** demonstrate low serum synergizes with chloroquine, but not due to lack of growth factors. (**G**) Summary of Snapshot Metabolomics of SW480 cells with control or Serum^hi^ or cotreated with vehicle or chloroquine. (**H**) MetaboAnalyst analysis of metabolites in Serum^hi^ with chloroquine. *P < 0.05, **P < 0.01, ***P < 0.001 using 2-way ANOVA with Tukey’s multiple-comparison test. Data are represented as mean ± SEM. All growth experiments were done in triplicate and repeated 3 times. The metabolomics were performed once in triplicates.

To understand if the dependency of autophagy in tumor cells is linked to cellular metabolic demands, we established a low dose of chloroquine or low-nutrient conditions that did not alter cell growth (Figure 5, A and B, and Supplemental Figure 6A). Cells cultured in a low-nutrient conditions in combination with low-dose chloroquine significantly decreased cell growth (Figure 5C) compared with either treatment alone. To understand the cellular metabolic demands that require autophagy, we heat-inactivated serum at 95°C (herein referred to as Serum^hi^) compared with the standard 52°C to remove heat-labile nutrients. Similar to reduced serum, Serum^hi^ combined with autophagy loss reduced cell growth (Figure 5D). Recent data in leukemic cell lines have shown that iron supplementation restores growth defects following lysosomal dysregulation (31). However, iron does not rescue growth defects in chloroquine-treated colon cancer cells (Supplemental Figure 6B). Moreover, to understand if low-dose chloroquine with glucose or iron depletion could recapitulate similar growth defects as Serum^hi^ combined with chloroquine, cells were treated in low glucose or iron media. Decreasing glucose or iron did not have an additive or synergistic effect on cell growth in combination with autophagy inhibition (Supplemental Figure 6, C and D). Moreover, supplementing insulin and epidermal growth factor (EGF) did not rescue the growth defect (Figure 5, E and F). The additive effect of autophagy loss with Serum^hi^ was similar following ULK1 inhibition (Supplemental Figure 7). To identify which metabolites were impacted under autophagy loss in combination with nutrient stress, the intracellular metabolomes of SW480 cells treated with Serum^hi^ or chloroquine at 2.5 μg/mL or cotreated with Serum^hi^ or chloroquine for 2 days were analyzed via liquid chromatography/mass spectrometry (LC/MS) (Figure 5G). This time point was selected as no change in growth is observed at 2 days (Figure 5D). Interestingly, we found only slight changes in the metabolome with either treatment alone, consistent with our growth data (Supplemental Table 1). However, cotreatment led to significant changes in several metabolites. Metabolites that were significantly changed in the Serum^hi^ and chloroquine group were analyzed for pathway analysis using MetaboAnalyst (32). A significant mitochondrial metabolite signature was found (Figure 5, G and H). However, supplementation of individual metabolites did not rescue the growth defects (Supplemental Figure 8). This suggests that a combination of metabolites is important in altering cell growth.

### CRC cells use mitophagy to meet cellular metabolic demands.

**Figure 6:**
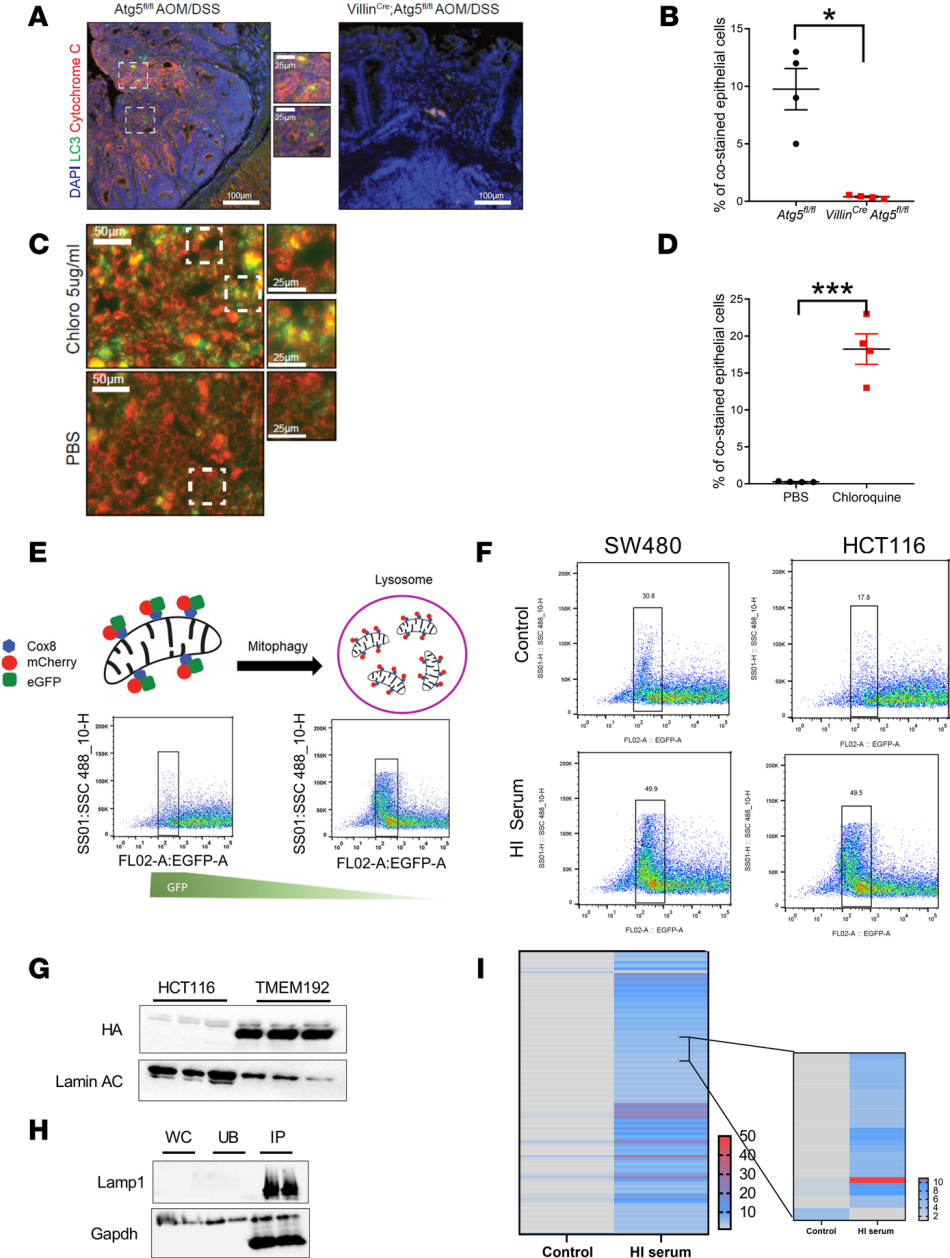
CRC cells employ mitophagy under nutrient stress. (**A** and **B**) Representative LC3 and cytochrome C costaining and quantitation of the staining in AOM/DSS-treated *Atg5^fl/fl^* and *Villin^Cre^*; *Atg5^fl/fl^*. (**C** and **D**) Costaining and quantitation of mito- and lyso-tracker in SW480 cells following 24 hours of treatment of 5 μg/mL of chloroquine. (**E**) Schematic of Cox8-mCherry-GFP flow cytometry. (**F**) Flow cytometry analysis of mitophagy following 2-day treatment with Serum^hi^ in SW480 and HCT116 cells. (**G**) Western blot confirmation of TMEM192-3xHA–expressing HCT116 cells. (**H**) Western blot of immunoprecipitation of TMEM192-3xHA cells in control or Serum^hi^. WC, whole cell lysate; UB, unbound fraction; IP, bound sample. Asterisk represents degraded GAPDH product. (**I**) Relative change in peptide spectral matches to total and mitochondria-specific proteins following treatment with Serum^hi^. All experiments were done in triplicate, and the proteomics were preformed once. *P < 0.05, ***P < 0.001 using unpaired *t* test. Data are represented as mean ± SEM.

Alterations in metabolites involved with the TCA cycle suggested an impact on mitochondria. Mitochondria can be targeted by autophagy through a process of selective autophagy known as mitophagy (33). Mitophagy as assessed by colocalization of LC3 and cytochrome C was significantly reduced in intestinal tissue from *Villin^Cre^*;*Atg5^fl/fl^* following AOM/DSS compared with *Atg5^fl/fl^* mice (Figure 6A). Similar data were observed in *Apc^fl/fl^*;*Atg5^fl/fl^* following tamoxifen treatment compared with *Apc^fl/fl^* mice (Supplemental Figure 9, A and B). Consistent with these data, SW480 and HCT116 treated with chloroquine have a robust increase in costaining using mitotracker and lysotracker, chemical reporters specific for mitochondria and lysosome, respectively (Figure 6, B and C, and Supplemental Figure 9, C and D). This suggests a dysregulation of mitochondrial degradation following chloroquine. Similar data were observed in enteroids, demonstrating a robust decrease in mitophagy following inhibition of macroautophagy (Supplemental Figure 9E). To assess if mitophagy is essential in CRC to meet the metabolic demands for proliferation, mitophagy flux was assessed in CRC-derived cell lines. The mitochondrial-specific protein cyctochrome c oxidase subunit 8 (COX8) fused to 2 fluorescent reporters, mCherry and GFP (COX8-mCherry-GFP), was used. If mitochondria are targeted to the lysosome, GFP fluorescence is quenched upon a change in pH, where mCherry fluorescence remains (Figure 6D) (34). Using flow cytometry, HCT116 and SW480 expressing Cox8-mCherry-GFP cultured in Serum^hi^ conditions demonstrated an increased flux in mitophagy following nutrient stress (Figure 6E). To further validate this observation, proteomic analysis was performed in lysosomes in control or Serum^hi^ conditions. A stable TMEM192-expressing HCT116 cell line was established to enrich for lysosomes via immunoprecipitation using a LysoIP method (Figure 6, F and G) (35). Lysosomal proteomics demonstrated an enrichment of mitochondrial proteins in the lysosome under Serum^hi^ (Figure 6, H and I, and Supplemental Table 2). The total lysosomal proteome content consisted of ~8% mitochondrial proteins, in which 90% of all mitochondrial proteins identified were higher in the lysosomes of Serum^hi^-treated cells. Together with the metabolomics analysis, this suggests that mitophagy is integrated with the cellular nutrient needs and is upregulated during nutrient stress.

### Mitophagy is essential for CRC growth.

**Figure 7:**
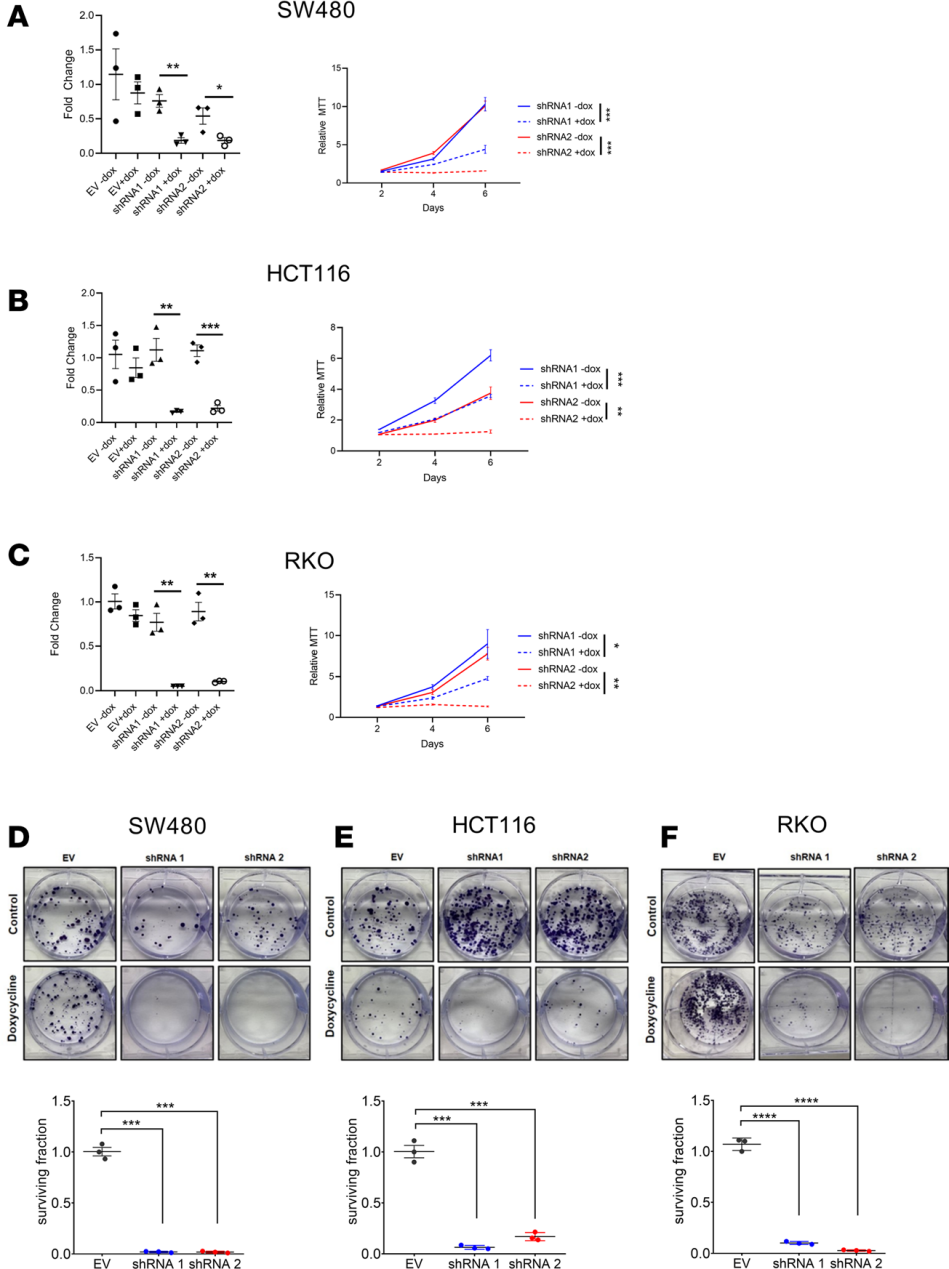
Mitophagy is necessary for CRC cell growth. (**A**) qPCR analysis of PINK1 shRNA knockdown and MTT assay in SW480. (**B**) qPCR analysis of PINK1 shRNA knockdown and MTT assay in HCT116. (**C**) qPCR analysis of PINK1 shRNA knockdown and MTT assay in RKO. *P < 0.05, **P < 0.01, ***P < 0.001, using 2-way ANOVA with Tukey’s multiple-comparison test. Data are represented as mean ± SEM. (**D**–**F**) Representative images of clonogenic assay and quantification by blinded observers in doxycycline-inducible shRNA specific for PINK1 (shRNA 1 and shRNA 2) or EV in SW480, HCT116, and RKO. ***P < 0.001, ****P < 0.0001 using 2-way ANOVA with Tukey’s multiple-comparison test. Data are represented as mean ± SEM. All experiments were done in triplicate and repeated 3 times.

To understand the contribution of mitochondrial targeting to the lysosome for CRC growth, mitophagy was genetically inhibited. PTEN-induced kinase 1 (PINK1) is important for inducing mitophagy (36). PINK1 is involved in PINK1/Parkin-mediated (PRKN-mediated) mitophagy and phosphorylates PRKN, which is then polyubiquitinated and targeted for autophagic degradation. We generated doxycycline-inducible shRNA constructs targeting *PINK1* in SW480, HCT116, and RKO cells (Figure 7, A–C). Knockdown of *PINK1* in these cell lines significantly reduced growth following doxycycline treatment, whereas empty vector was not changed, as assessed by MTT (Figure 7, A–C, and Supplemental Figure 4A) and long-term clonogenic cell survival assays (Figure 7, D–F). Moreover, iron was not able to rescue the growth phenotype in *PINK1* KD cells, further suggesting that lysosomal function is not needed to maintain iron homeostasis (Supplemental Figure 10, A and B). Similarly, knockdown of *PRKN* in HCT116 cells significantly reduced growth when assessed by clonogenic assay (Supplemental Figure 10, C–E).

## Discussion

Autophagy is a cellular process that allows for the sequestration and breakdown of organelles and cellular components. Autophagy is found to be both pro- and antitumorigenic (37–39). Heterocellular cross-talk exists between tumor epithelium and the microenvironment, and current work in CRC mouse models demonstrates an important role of epithelial autophagy in sustaining an immunosuppressive environment via gut commensals (7). Importantly, the activation of autophagy in CRC is found to be context dependent on microbial infiltration, inflammation, and tumor stage (7, 40–43). While autophagy is often thought to be a mechanism for nutrient recycling, or degradation of dysfunctional organelles, the precise role in CRC is not known. Moreover, the role of autophagy in maintaining nutrient pools in CRC as not been definitively assessed. Specifically, the metabolic cues that activate autophagy, and the cellular metabolites that autophagy provide to maintain growth, have not been investigated in CRC. We have shown that loss of autophagy through ATG5 inhibits tumor growth in a cell-autonomous fashion in inflammation-driven (AOM/DSS), sporadic (*Apc*), and patient-derived in vitro models of CRC. Mechanistically, we show that — under nutrient stress — autophagy is directly integrated to meet nutrient demands via mitophagy.

We observed no changes in immune cell infiltration or immune signaling as previously described (7). Differences may be attributed to experimental design. For our experiments, we use littermate controls and have standardized the microbiome by mixing the bedding prior to tumor induction to prevent potential microbiota differences (44). It is also documented that microbiota differ based on housing facilities (45). While other studies have identified immune differences, our experimental design and potential microbial differences allowed us to highlight the cell-autonomous role of autophagy in tumor development.

The hyperproliferative nature of tumor cells reprograms cellular metabolism and activate pathways to replenish nutrient pools in tumor cells. In pancreatic cancer, cells scavenge for extracellular proteins to acquire amino acids (46). Breast cancer utilizes autophagy under starvation to maintain amino acid levels (47). In our study, we have identified autophagy as a key function that CRC cells rely on for proliferation. Our in vitro cell models are cultured in a highly nutrient-rich medium. Upon a challenge with pharmacological or genetic autophagy inhibition, growth is dramatically reduced. This suggests that colon cancer cells are addicted to autophagy for growth and have adapted to rely on this mechanism for proliferation, even in the context of available nutrients. To integrate autophagy to cellular metabolic demands, we found that loss of heat-labile nutrients in serum (but not iron or glucose) led to a robust decrease in cell growth, in combination with autophagy inhibition. However, these results suggest that nutrients acquired through autophagy — and, more specifically, mitophagy — are required for general cell maintenance in tumors. This is supported by the basal levels of mitophagy that we observed in colon cancer cells under nutrient-rich culture conditions. Recent work has demonstrated that iron is the key nutrient to maintain growth after lysosomal inhibition in a battery of cell lines (31). Colon cancer–derived cell lines were not rescued by iron following lysosomal inhibition. Intestinal cell lines are unique and can uptake iron via both apical and basolateral mechanisms. Moreover, intestinal cell lines have a robust expression of iron regulatory proteins that can rapidly alter ferritin translation and sustain intracellular iron levels (48). While we were unable to rescue growth with individual supplementation of nutrients, it is possible that the combination of nutrients acquired through mitophagy is required for cell proliferation. While we attempted to assess metabolites in pathways that were significantly affected, it is possible that an individual, undetermined metabolite contributes to the observed changes in cell growth. The metabolomics data, the rapid decrease in cell growth when autophagy and mitophagy are inhibited, and a potentiation of reduced cell growth in combination with nutrient stress suggest that a major role of mitophagy is to replenish the nutrient pool in cancer cells. However, a decrease in growth could also be due to reduced recycling of defective mitochondria. Moreover, autophagy is essential in regulation of proteins critical for cell growth (49). Future work is focused on decoupling the importance of nutrient recycling to other autophagic functions in colon cancer cell growth.

To clearly understand the role of autophagy in CRC, patient-derived enteroid models and adjacent normal enteroids were utilized. Patient-derived tumor enteroids (28) treated with chloroquine showed a marked decrease in growth when compared with patient-derived normal enteroids. The tumor selective response further highlights the essential role of autophagy modulation in tumor growth. Interestingly, we observed no growth inhibition in the BRAF mutant (Val600Glu) enteroid model. *BRAF* mutations are present in about 10% of patients (50). This particular enteroid was generated from a sessile serrated tumor (28), and *BRAF* mutations are known to be drivers for this tumor type (29, 30). We are not aware of any literature that investigates the functional role of autophagy in sessile tumors, but this finding uncovers the importance of understanding autophagy under different mutational burdens. Furthermore, it is important to consider the mutational load present within the models used in our study and others. p53 is mutated in about 50% of CRCs (50). However, the *Atg7* model discussed above (7) and our AOM/DSS and sporadic tumor models typically do not harbor p53 mutations (51, 52). Extensive work is needed to understand the genotypic variability in CRC to autophagy inhibition.

We have identified mitophagy as an important selective pathway for nutrient acquisition in colon tumors. Mitophagy is a newly studied modulator of cancer growth, and its particular role in CRC is not well understood. A study identified DNA copy number loss of PRKN (*PARK2* gene) in about 33% of the colon tumors screened. *PRKN* deletion enhanced tumor growth in *Apc^+/Min^* mice. In addition to PRKN being important in mitophagy, PRKN is an E3 ubiquitin ligase for cyclin E. Loss of PRKN led to an increase in cyclin E and progression of the cell cycle (53). The use of pharmacological tools to activate mitophagy are already in development for cancer treatments. In *KRAS* mutant CRC, treatment with pharmacological inhibitors of mitochondria, Mito-CP, and Mito-Met_10_ increase mitophagy and decrease cell proliferation (54). The data are consistent with work showing that overexpression of PINK1 can lead to increase in cancer cell growth (55). Moreover, mitophagy in tumor epithelium was shown to activate CD8^+^ T cells to reduce tumor burden in the colon (56). The cell-autonomous role of mitophagy was not directly assessed on cell growth. Here, our work outlines a role for PINK/PRKN-mediated mitophagy in an immune cell–independent context. Moreover, our data suggest that, under nutrient stress, mitophagy is required to sustain metabolite pools for growth. Clinically, the expression of PRKN is prognostic in patient outcome. Decreased PRNK expression is correlated with increased survival (57); however, increased expression is found with enhanced invasion in tumors (57). Our work highlights that the dual nature of autophagy and mitophagy in cancer growth could be attributed to available nutrient status. Clearly, more studies are needed in a large panel of cancer cell lines to understand if the mutational landscape dictates a pro- or antitumor response of mitophagy. It is also important to consider that PINK1-PRKN–independent mechanisms of mitophagy exist (58–60).

This work underscores the importance of autophagy in nutrient acquisition in CRC and the potential for mitophagy inhibition to be used alone or in combination with other chemotherapeutics to improve overall CRC outcomes.

## Methods

### Mouse experiments.

For all experiments, male and female mice at 6–8 weeks of age were used. All mice were bred on a C57BL/6 background. ATG5 TM1a conditional embryonic stem cells were acquired from Riken, and the mice were generated by the University of Michigan Transgenic core. The microbiome was normalized for 1–2 weeks prior to experiment initiation by combining bedding and distributing it evenly among experimental mice. DSS experiments were completed by placing mice on 2.0% DSS in water for 7 days followed by a 3-day recovery on regular drinking water. For AOM/DSS experiments, mice were injected i.p. with 10 mg/kg of AOM. Five days after injection, mice were cycled on and off 2.0% DSS in their drinking water for 1 week, followed by a 2-week recovery as previously described (61). Weights were taken daily. For spontaneous tumors (*Cdx^ERT2^*;*Apc^fl/fl^*;*Atg5^fl/fl^*), mice were injected with a single dose (50 mg/kg) of tamoxifen. Six weeks later, tissue was collected. Tumor burden is a summation of total tumor volume per mouse.

### Histology and immunofluorescence.

Histological analysis was scored by a blinded pathologist as previously described (62). Tissues were collected and fixed in 10% formalin for 24 hours, followed by embedding in paraffin. Sections (5 μM) were stained for H&E. Immunofluorescence of Ki67 (1:100; Cell Signaling Technology, 12202) was completed using antigen retrieval in sodium citrate (Tri-sodium citrate 11.4 mM, pH 6.0, 0.05% Tween-20) and labeled with Alexa Fluor 488 (Thermo Fisher Scientific, A11008). Tissue was mounted with ProLong Gold with DAPI (Invitrogen). Images were quantified using ImageJ software (NIH) as percent of Ki67^+^ area to DAPI^+^ area. Mitotracker and lysotracker in colon cancer cell lines were assessed in 6-well plates and grown for 1 day with 5 μg/mL chloroquine or PBS. The next day, fresh media containing 75 nM Lysotracker Green DND-26 (Thermo Fisher Scientific, L7526) was added for 15 minutes. This media was removed and replaced with media containing 75 nM Lysotracker and 100 nM Mitotracker Red CMXRos (Thermo Fisher Scientific, M7512) for 20 minutes. Media was replaced with PBS, and cells were immediately imaged. Mitophagy and autophagy in tumor tissue were prepared for immunofluorescence following deparaffinization and rehydration protocols. Antigen retrieval was performed in sodium citrate buffer with 0.05% Tween 20 at pH 6. Slides were blocked for 15 minutes in PBS containing 0.2% Triton X-100 (Thermo Fisher Scientific) and 2.5% BSA; they were then blocked for 1 hour in MoM Blocking Reagent (Vector Labs, MKB-2213-1). Primary antibodies were incubated overnight in blocking buffer (mouse anti–cytochrome C [1:250], Abcam, ab13575; rabbit anti-LC3B [1:250], Novus, NB600-1384). Sections were washed in PBS and incubated for 1 hour with fluorescent secondary antibodies and washed prior to mounting.

### RNA isolation and qPCR analysis.

RNA was isolated using TRIzol chloroform extraction. RNA was reverse transcribed using MMLV reverse transcriptase (Thermo Fisher Scientific). Quantitative PCR (qPCR) analysis was done using the listed primers (Supplemental Table 3) and Radiant Green qPCR master mix (Alkali Scientific Inc.). For cell isolation of epithelial cells and immune cells, EpCAM and CD45 mouse microbeads were used prior to RNA isolation (Miltenyi Biotec).

### Enteroid culture.

Enteroids were cultured as previously described (28). Lines 87 and 89 were cultured in completed L-WRN medium. Additional lines (lines 282, 584, 590, 245) were cultured in Kerotinocyte Growth Medium (Thermo Fisher Scientific). Cultures were plated in Matrigel (Corning) and allowed to establish for at least 3 days. Following establishment, cells were treated either with control (Sterile PBS) or chloroquine at 75 μg/mL (in PBS) for 3 days. Images were taken at 24 and 72 hours after treatment. Measurements were completed by normalizing the relative area of an individual enteroid to day 0. All measurements were completed by a blinded observer.

### Flow cytometry.

For Cox8-mCherry-eGFP, Bio-Rad Ze5 Cell Analyzer was used. Cells were first sorted for mCherry positivity followed by eGFP. Analysis was done using FlowJo software. Flow cytometry analysis of immune cells was done using the Beckman Coulter MoFlo Astrios; immune cells from the colon were isolated by 25 mM EDTA digestion to remove epithelial cells, followed by a 0.5 mg/mL collagenase IV digestion, and were enriched for using a 40%–70% percoll gradient. Immune cells were stained for with CD45 APC eFluor 780, 1:200 (Invitrogen 47-0451-82); CD4 PECy7, 1:300 (eBioscience, 25-0041-82); CDllc FITC, 1:200 (BioLegend, 117305); CDllb APC, 1:250 (eBioscience, 17-0112-83); Ly6C V450, 1:300 (BD Biosciences, 560594), Ly6G PE, 1:300 (BD Biosciences, 560594), F4/80 BV510, 1:100 (BD Biosciences, 563633), 7AAD Percp Cy 5.5, 1:300 (BD Biosciences, 559925).

### MTT assays.

Twenty-four hours following plating, a day 0 reading was taken. Cells were incubated for 45 minutes with Thiazolyl Blue Tetrazolium Bromide (MilliporeSigma). Then, they were solubilized with dimethyl sulfoxide. Absorbance was read at 570 nm. Following the day 0 read, the corresponding treatment and readings were taken every 24 hours for a 72-hour assay or every other day for a 6-day assay. All reads were taken in technical triplicates.

### Protein isolation and Western blotting.

All protein samples were separated by SDS-PAGE and transferred on to the nitrocellulose membrane. Antibodies were used as follows: TFEB, 1:1000 (Bethyl, A303-672A-M); LC3B, 1:1000 (Cell Signaling Technology, 2775S); ATG5, 1:1000 (Santa Cruz Biotechnology Inc., sc-133158); HA-Tag, 1:1000 (Abcam, 18181); LAMP1, 1:1000 (Cell Signaling Technology, 9091S); Lamin AC, 1:1000 (Active Motif, 39287); GAPDH, 1:1000 (Santa Cruz Biotechnology Inc., 47724); β-actin, 1:1000 (Proteintech, 66009-1-Ig); and PRKN, 1:1000 (Cell Signaling Technology, 4211).

### Cell lines.

All cell lines were cultured in DMEM with 10% FBS unless otherwise noted. Stable *TFEB*-KO line was generated using gRNA in Lenticrispr V2 (Addgene plasmid 49535) (63) using the guides listed (Supplemental Table 3). Constructs for doxycycline-inducible shRNA were generated using the Tet-pLKO-puro (Addgene plasmid 21915). Plasmids were generated and inserted in to a lenti-viral vector for stable transfection. Knockdown was induced using 200 ng/mL of doxycycline for 48 hours. The HCT116 cells used for tracking mitophagy were generated from the pCLBW Cox8-mCherry-EGFP plasmid (Addgene plasmid 78520). ATG4B mutant–expressing cell line was developed by stable expression of pmStrawberry-Atg4B^C74A^ (Addgene plasmid 21076). We generated the HCT116 LysoIP line using the pLJC5-Tmem192-3xHA (Addgene plasmid 102930). Cells were treated chloroquine diphosphate (MilliporeSigma) and SBI-0206965 (Cayman Chemical) using concentration and time as shown in the figure.

### Metabolomics.

Polar metabolites were extracted in ice cold 80% methanol on dry ice for 10 minutes. Proteins and cell debris were precipitated by centrifugation at 15,000*g* for 10 minutes at 4°C. Metabolite supernatants were dried on a SpeedVac and submitted for steady state metabolomics profiling (64, 65). An Agilent 1290 Infinity II LC -6470 Triple Quadrupole (QqQ) MS/MS system was used. For negative ion acquisition, a Waters Acquity UPLC BEH amide column (2.1 *×* 100 mm, 1.7 μm) was used with the mobile phase A consisting of 97% water, 3% methanol, 10 mM tributylamine, 15 mM acetic acid, and 5 μM Agilent infinity lab deactivator additive and mobile phase B, consisting of 10mM tributylamine, 15mM glacial acetic acid, and 5 μM Agilent infinity lab deactivator additive. Pump A and C deliver buffer A and B, respectively. Pump D delivers acetronitrile to wash the column at the end of the run. The following gradient was used: 0–2.5 minutes, 100% A at 0.25 mL/min (27 minutes for the analytical run); at 7.5 minutes, 80% A; at 13 minutes, 55% A; at 20 minutes, 1% A and kept to 24.0 minutes; at 24.05–27 minutes, 1%A and 99% D; at 27.05–31.35 minutes, 1% A and 99% D at 0.8 mL/min flow rate; at 32.25–39.9 minutes, 100% A at 0.40 mL/min flow rate; and at 40 minutes, 100% A, 0.25 mL/min. The column was kept at 40°C, and 3 μL of sample was injected into the LC-MS/MS with a flow rate of 0.2 mL/min. Tuning and calibration of the QqQ was achieved through Agilent ESI Low Concentration Tuning Mix.

The MassHunter Metabolomics Dynamic MRM Database and Method was used for target identification. Key parameters of AJS ESI were: gas temperature, 150°C; gas flow, 13 L/min; nebulizer, 45 psi; sheath gas temperature, 325°C; sheath gas flow, 12 L/min; capillary, 2000 V; and nozzle, 500 V. Detector Delta EMV(-) 200.

The QqQ data were preprocessed with Agilent MassHunter Workstation Quantitative Analysis Software (B0700). Each metabolite was median normalized across all samples for proper comparisons, statistical analyses, and visualizations among metabolites. The statistical significance test was done by a 2-tailed *t* test with a significance threshold level of *P* < 0.05.

### Proteomics.

Cells were kept in control or media with Serum^hi^ for 6 days. Cell were lysed, and lysosomes were isolated as previously described (35) with anti-HA tag (Thermo Fisher Scientific, 88836). Beads were washed twice with TBS-T and twice with PBS. The beads were resuspended in 50 mL of 0.1M ammonium bicarbonate buffer (pH 8). An overnight digestion with 1 μg sequencing grade, modified trypsin was carried out at 37°C with constant shaking in a Thermomixer. Digestion was stopped by acidification, and peptides were desalted using SepPak C18 cartridges using manufacturer’s protocol (Waters). Samples were completely dried using vacufuge. Resulting peptides were dissolved in 8 mL of 0.1% formic acid/2% acetonitrile solution, and 2 mL of the peptide solution were resolved on a nanocapillary reverse phase column (Acclaim PepMap C18, 2 μm, 50 cm; Thermo Fisher Scientific) using a 0.1% formic acid/2% acetonitrile (Buffer A) and 0.1% formic acid/95% acetonitrile (Buffer B) gradient at 300 nL/min over a period of 180 minutes (2%–25% buffer B in 110 minutes, 25%–40% in 20 minutes, and 40%–90% in 5 minutes, followed by holding at 90% buffer B for 10 minutes and requilibration with Buffer A for 30 minutes). Eluent was directly introduced into Q exactive HF MS (Thermo Fisher Scientific) using an EasySpray source. MS1 scans were acquired at 60K resolution (AGC target, 3 *×* 10^6^; max ion trap [IT], 50 ms). Data-dependent collision–induced dissociation MS/MS spectra were acquired using Top speed method (3 seconds) following each MS1 scan (normalized collision energy [NCE], 28%; 15K resolution; automatic gain control [AGC] target, 1 *×* 10^5^; max IT, 45 ms).

Proteins were identified by searching the MS/MS data against UniProt H Sapiens database (20331 entries; downloaded on 12/04/2018) using Proteome Discoverer (v2.1, Thermo Fisher Scientific). Search parameters included MS1 mass tolerance of 10 ppm and fragment tolerance of 0.2 Da; 2 missed cleavages were allowed. Carbamidimethylation of cysteine was considered fixed modification and oxidation of methionine, and deamidation of aspergine and glutamine were considered as potential modifications. FDR was determined using Percolator, and proteins/peptides with a FDR of ≤ 1% were retained for further analysis. Samples were normalized to the unbound fraction, and relative peptide spectral matches were compared between control and Serum^hi^.

### Statistics.

Statistical analyses were calculated by unpaired 2-tailed *t* test or 1- or 2-way ANOVA Tukey’s multiple-comparison test. Data are represented as mean ± SEM Data are represented as mean ± SEM.

### Study approval.

All animal studies were reviewed and approved by the IACUC at the University of Michigan in Ann Arbor Michigan.

## Author contributions

YMS and SND conceived the project. SND, RS, JGT, SAK, JWG, BC, MDR, CAL, CJ, NKD, LZ, and JKG performed experiments and analyzed data. SAS, KOW, YEC, and PR provided critical reagents for the study. YMS, SND, and RS wrote the manuscript with critical input from all authors.

## Supplementary Material

Supplemental data

Supplemental Table 1

Supplemental Table 2

Supplemental Table 3

## Version 1 06/17/2021

In-Press Preview

## Version 2 07/22/2021

Electronic publication
