# Supplementary material for: Colorectal cancer cells utilize autophagy to maintain mitochondrial metabolism for cell proliferation under nutrient stress
Source: JCI Insight. 2021 Jun 17;6(14):e138835. doi: 10.1172/jci.insight.138835 (PMC8328084; doi:10.1172/jci.insight.138835)
Supplement: Supplemental data [file jciinsight-6-138835-s008.pdf]

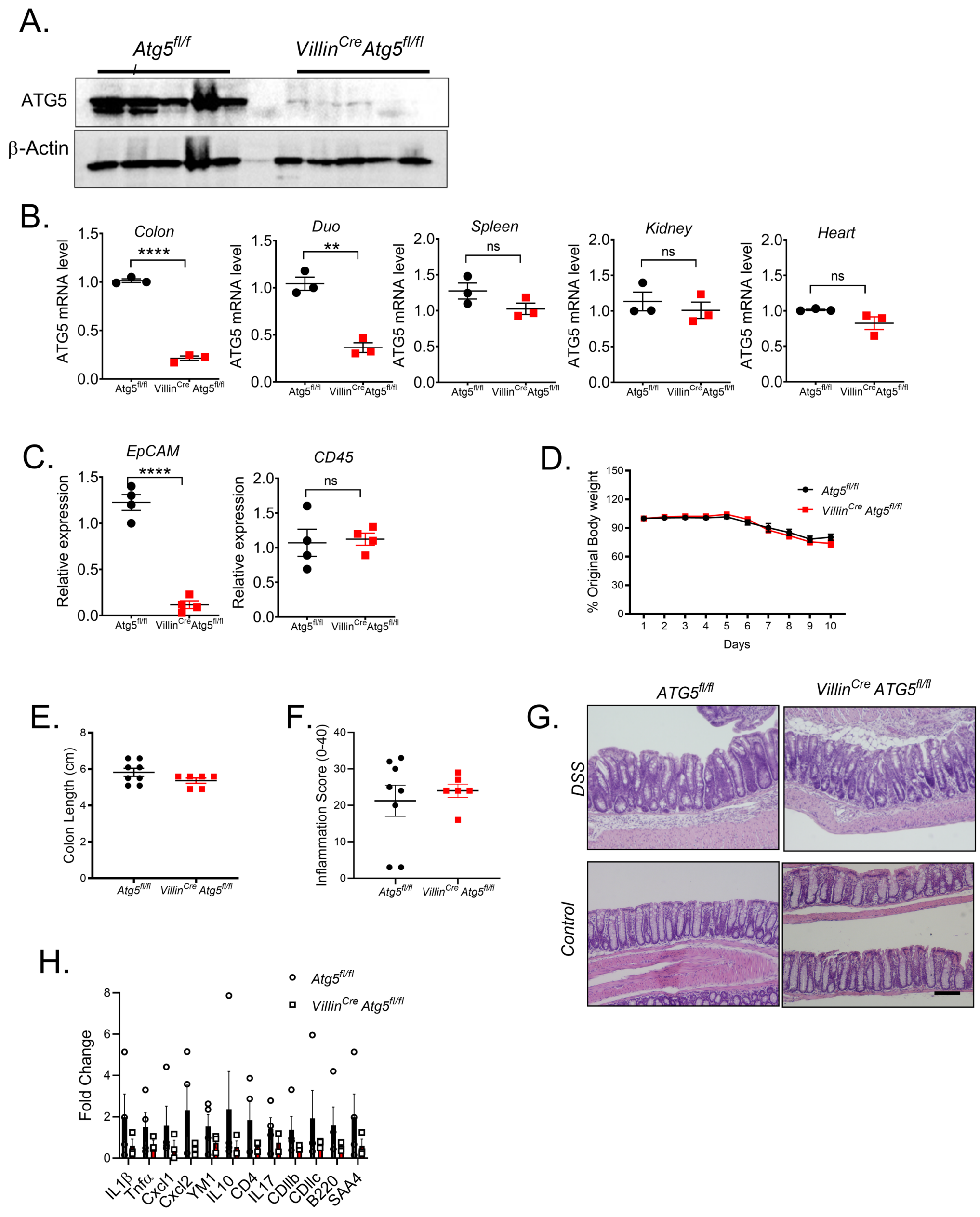

Figure S1 Autophagy loss does not impact acute colitis. A. Western blot of *Atg5$^{fl/fl}$* and *Villin^{Cre}; Atg5$^{fl/fl}$* mice in intestinal tissue lysates. ATG5 gene expression **B.** in tissues (colon, duodeneum (Duo), spleen, Kidney, and Heart) or purified EpCAM+ or CD45+ cells from the colons of *Atg5$^{fl/fl}$* and *Villin^{Cre}; Atg5$^{fl/fl}$* mice. **D.** Weights, **E.** colon length, and **F.** inflammation score and **G.** histological images of *Atg5$^{fl/fl}$* and *Villin^{Cre}; Atg5$^{fl/fl}$* mice following 7-day DSS with 3 day recovery. **H.** Panel of cytokines and chemokines following DSS in *Atg5$^{fl/fl}$* and *Villin^{Cre}; Atg5$^{fl/fl}$*. **** p < 0.001, **p <0.01 using unpaired t-test. Error bars represent SEM.

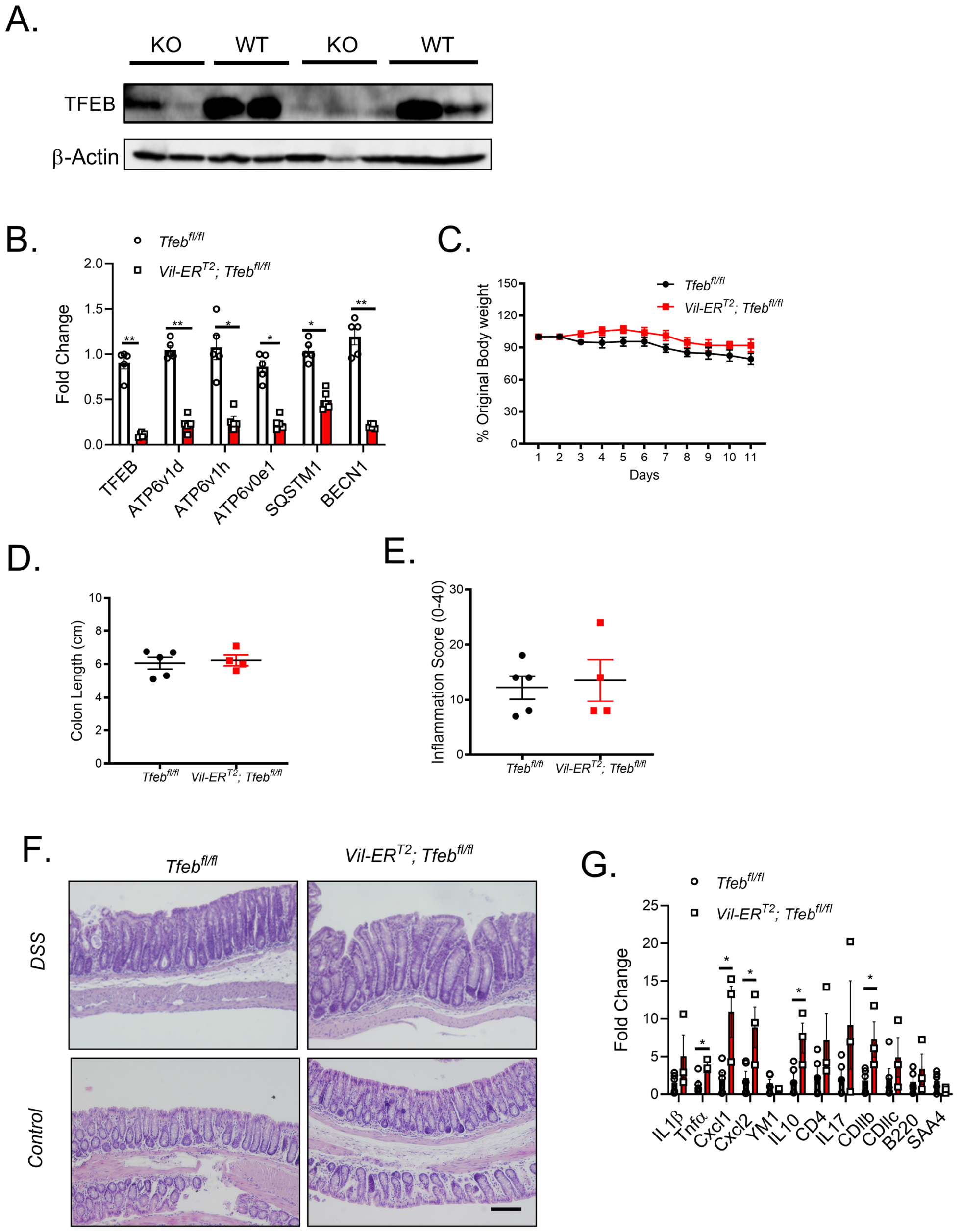

Figure S2 TFEB disruption does not impact acute colitis. A. Western blot of TFEB and **B.** lysosome and autophagy target genes *Tfeb*$^{fl/fl}$ and *Vil-ER*^{T2} mice. **C.** Weights, **D.** colon length, and **E.** inflammation score and **F.** H&E staining of control versus DSS treated *Tfeb*$^{fl/fl}$ and *Vil-ER*^{T2}*; Tfeb*$^{fl/fl}$ mice following 7-day DSS with 3 day recovery. **G.** Panel of cytokines and chemokines following DSS in *Tfeb*$^{fl/fl}$ and *Vil-ER*^{T2}. **L.** ** p < 0.01, *p <0.05 using unpaired t test. Error bars represent SEM.

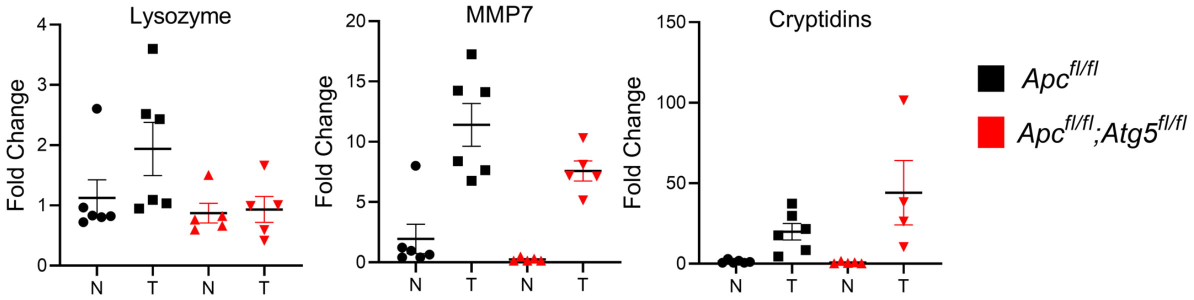

Figure S3 Paneth cell are not altered following disruption of ATG5 in a sporadic colon cancer model. qPCR analysis of Paneth cell markers in *Cdx2-ER*^{T2}Cre;*Apc*$^{fl/fl}$ or *Cdx2-ER*^{T2}Cre;*Apc*$^{fl/fl}$;*Atg5*$^{fl/fl}$ following 6 weeks of tumor formation. N- normal tissue, T- tumor tissue. Error bars represent SEM.

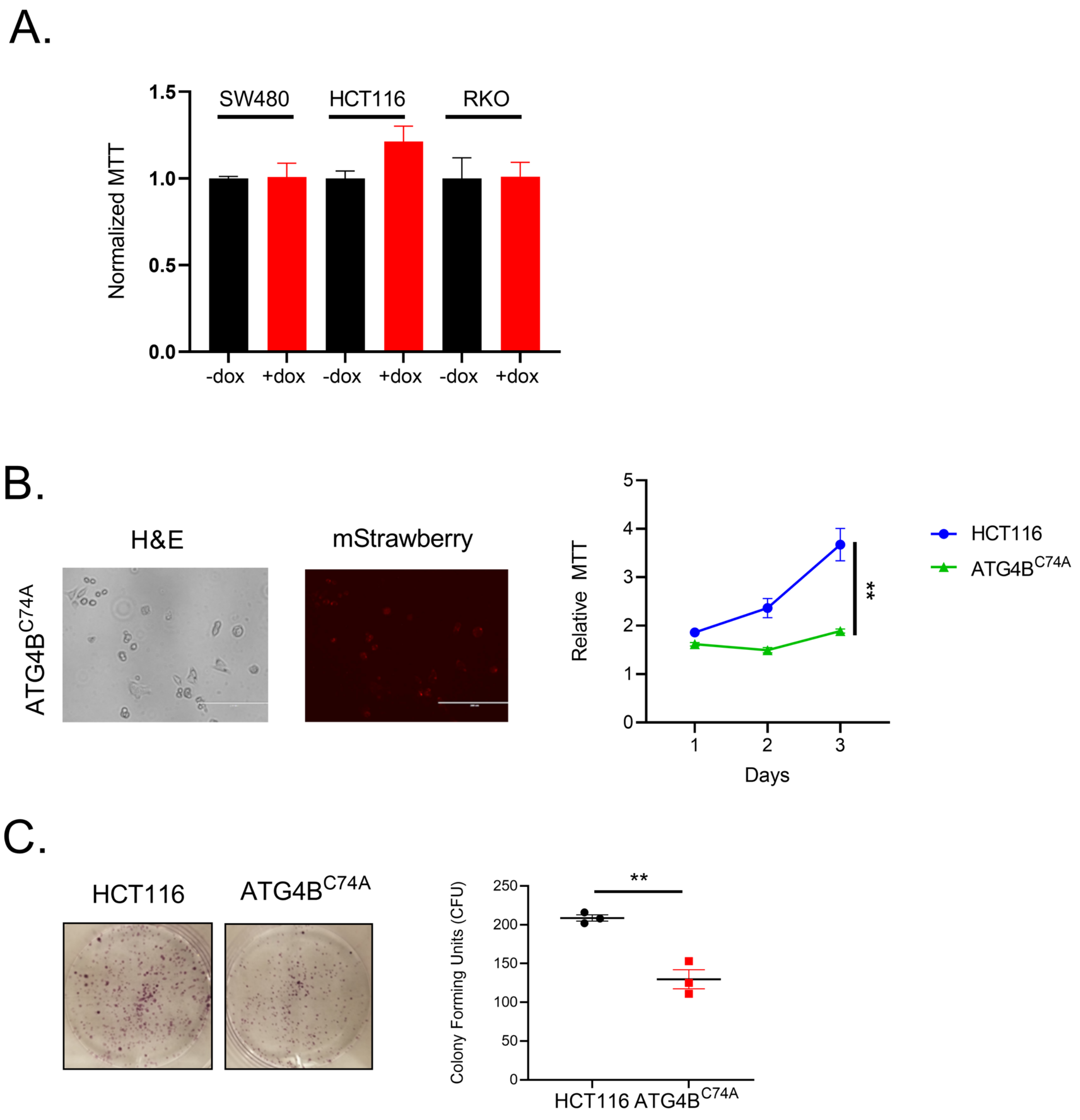

Figure S4 ATG4B inhibition reduces CRC growth. A. Analysis of empty vector constructs for doxycycline (dox) inducible shRNA constructs treated with doxycycline for 6 days. **B.** Image of mStrawberry expressing HCT116 ATG4B^{C74A} expressing cells and MTT assay of HCT116 ATG4B^{C74A} mutant. **C.** Representative image and quantification of clonogenic assay for ATG4B^{C74A} HCT116 cells. Scale bar 200μm. ** $p<0.01$ using unpaired t-test. Error bars represent SEM.

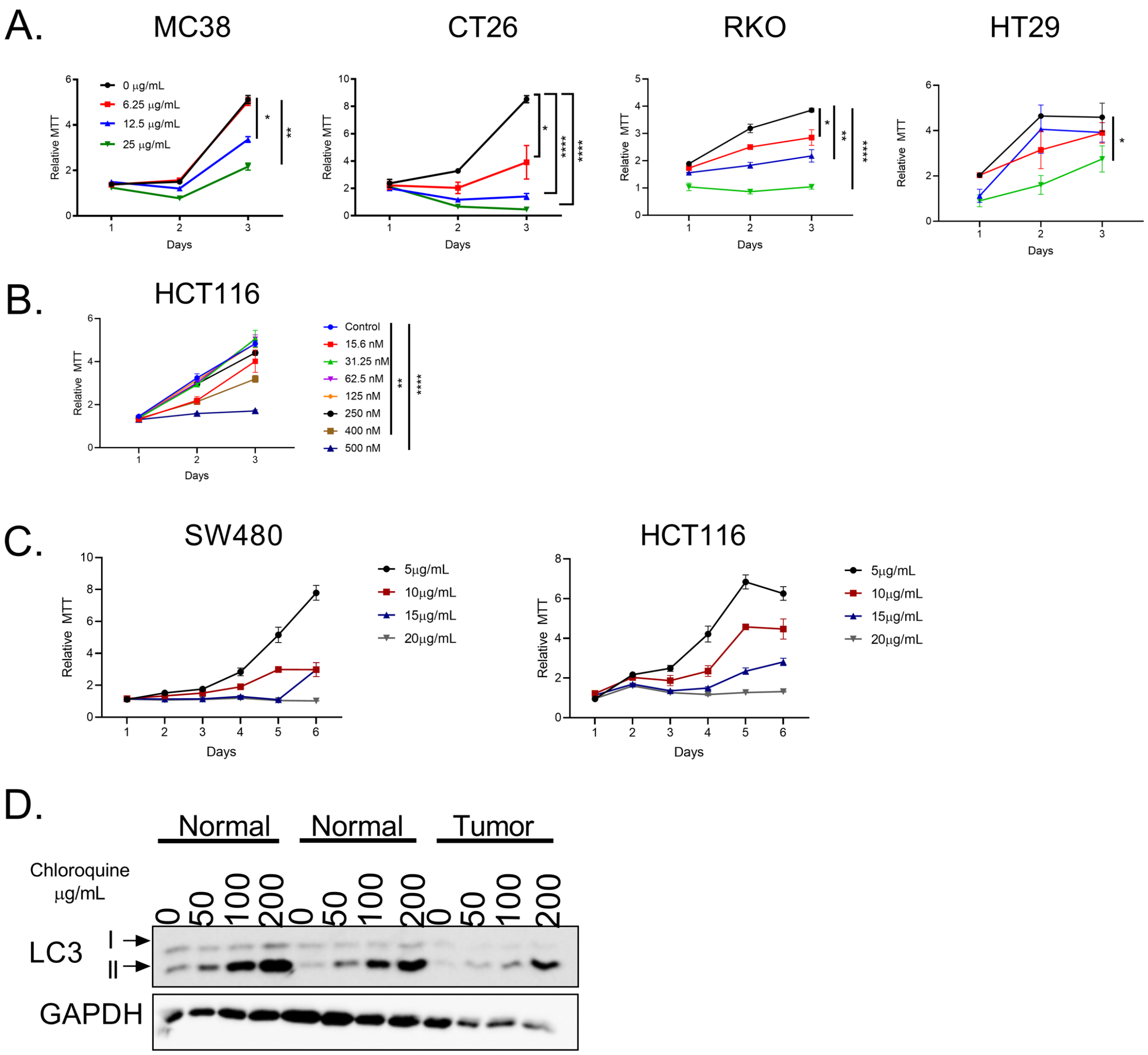

Figure S5 CRC cells are sensitive to pharmacological autophagy inhibition but do not induce apoptosis A. MTT assay upon treatment with chloroquine in MC38, HT29, CT26, and RKO. **B.** MTT assay upon treatment with SBI-0206965 in HCT116 cells. **C.** MTT assay of HCT116 and SW480 cells treated for 3-days with chloroquine and measured for 3-days after removal of chloroquine. **D.** Western blot of LC3 expression in patient derived enteroids. * $p<0.05$, ** $p<0.01$, **** $p<0.0001$ using two-way ANNOVA with Tukey's multiple comparison test. Error bars represent SEM.

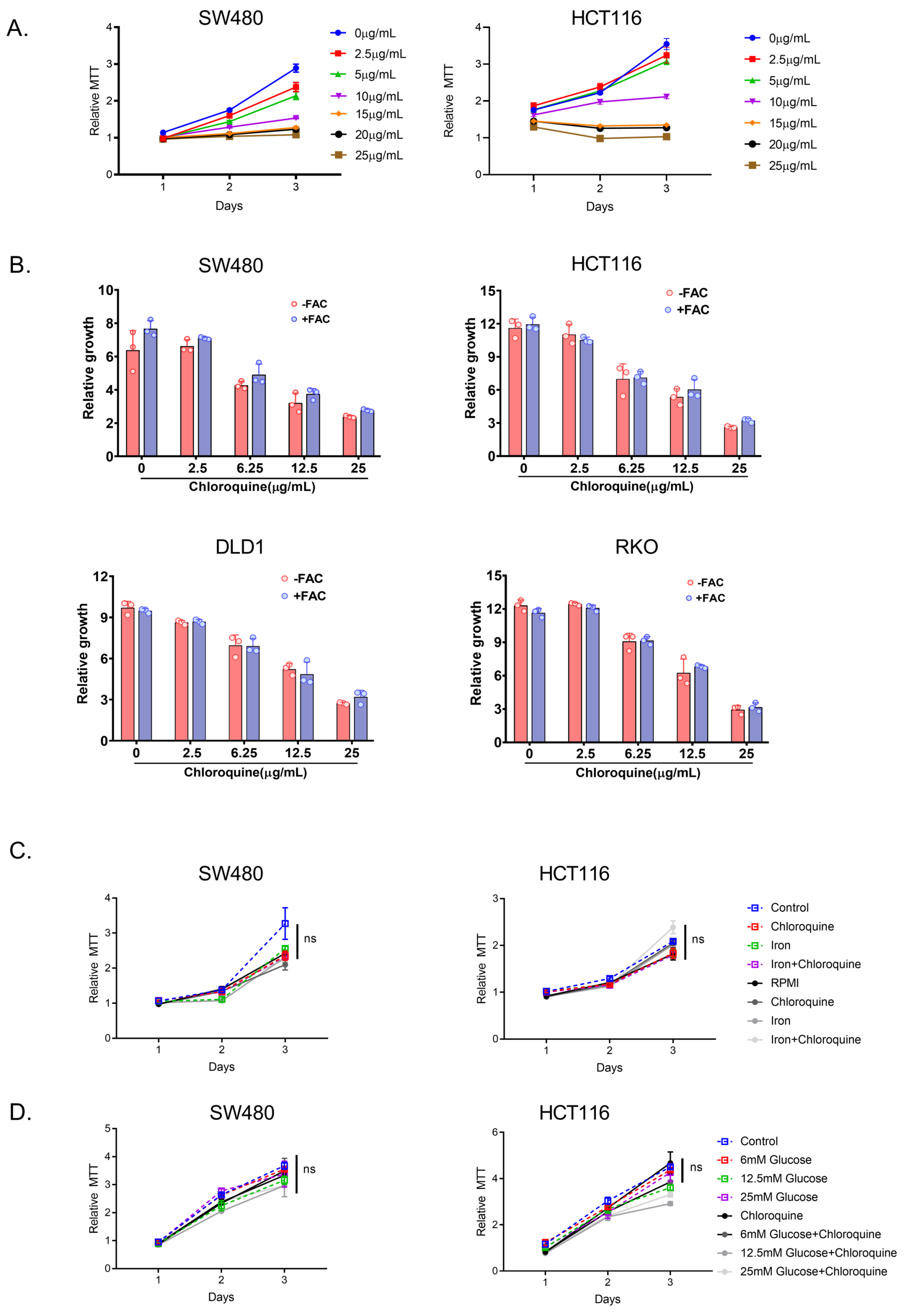

Figure S6 Nutrient challenge of iron or glucose loss with autophagy inhibition does not slow cancer cell growth. A. MTT dose response of chloroquine treatment in SW480 and HCT116 cells. **B**. MTT growth assay after 72 hours with following co-treatment with chloroquine and iron (FAC 100μM). **C.** MTT in low-iron media and rescue with iron. **D.** MTT in glucose free media and rescue with glucose. Error bars represent SEM.

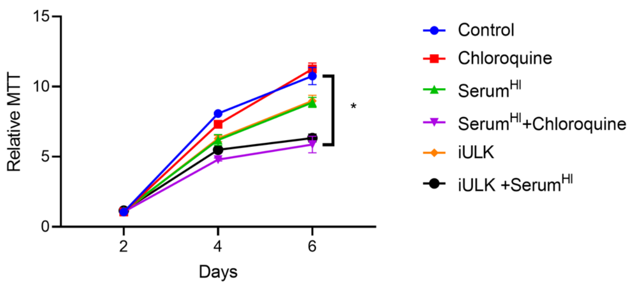

Figure S7 ULK inhibition decreases CRC growth. A. MTT of SBI-0206965 (iULK) treatment with Serum^{HI}. * $p < 0.05$. Error bars represent SEM.

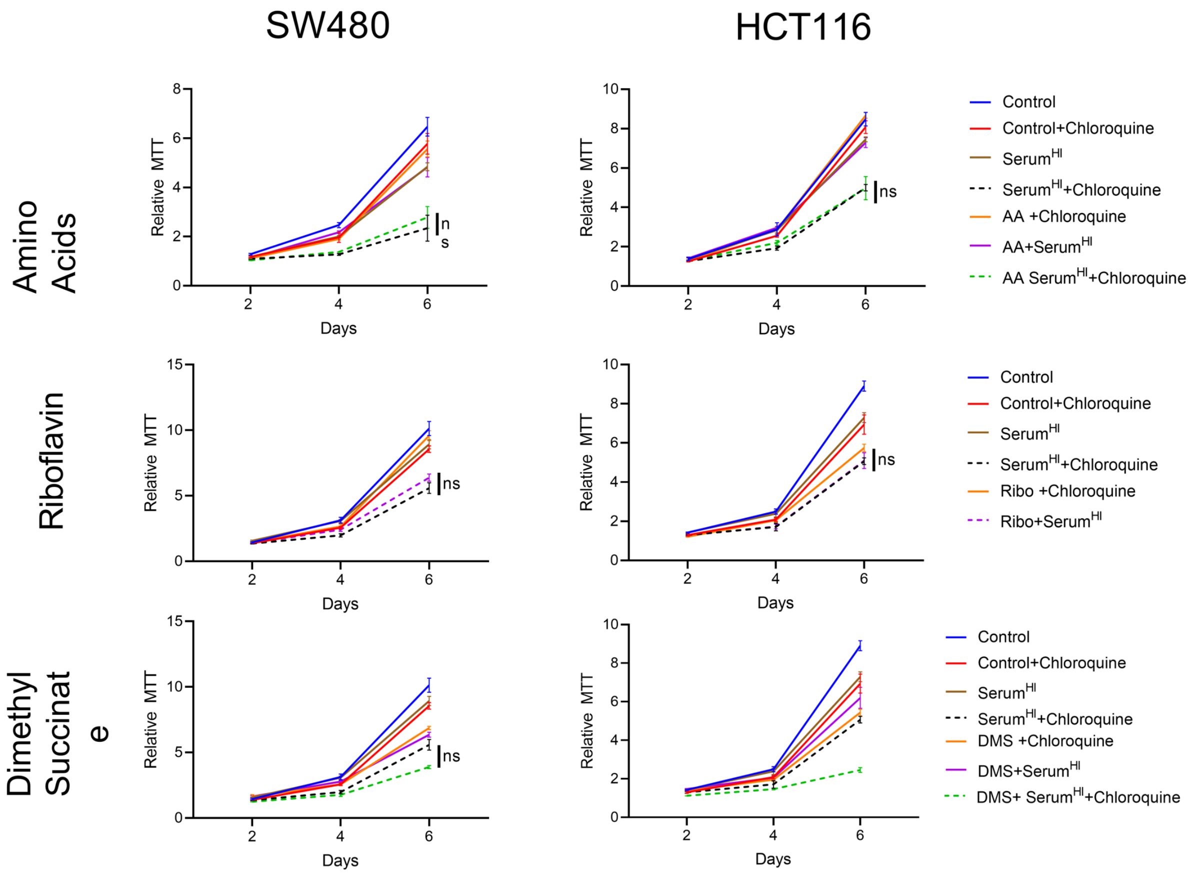

Figure S8 Metabolite supplementation under Serum^{HI} and chloroquine does not rescue growth. MTT assay of rescue with amino acids, riboflavin, and dimethyl succinate following treatment with Serum^{HI} with chloroquine. Error bars represent SEM.

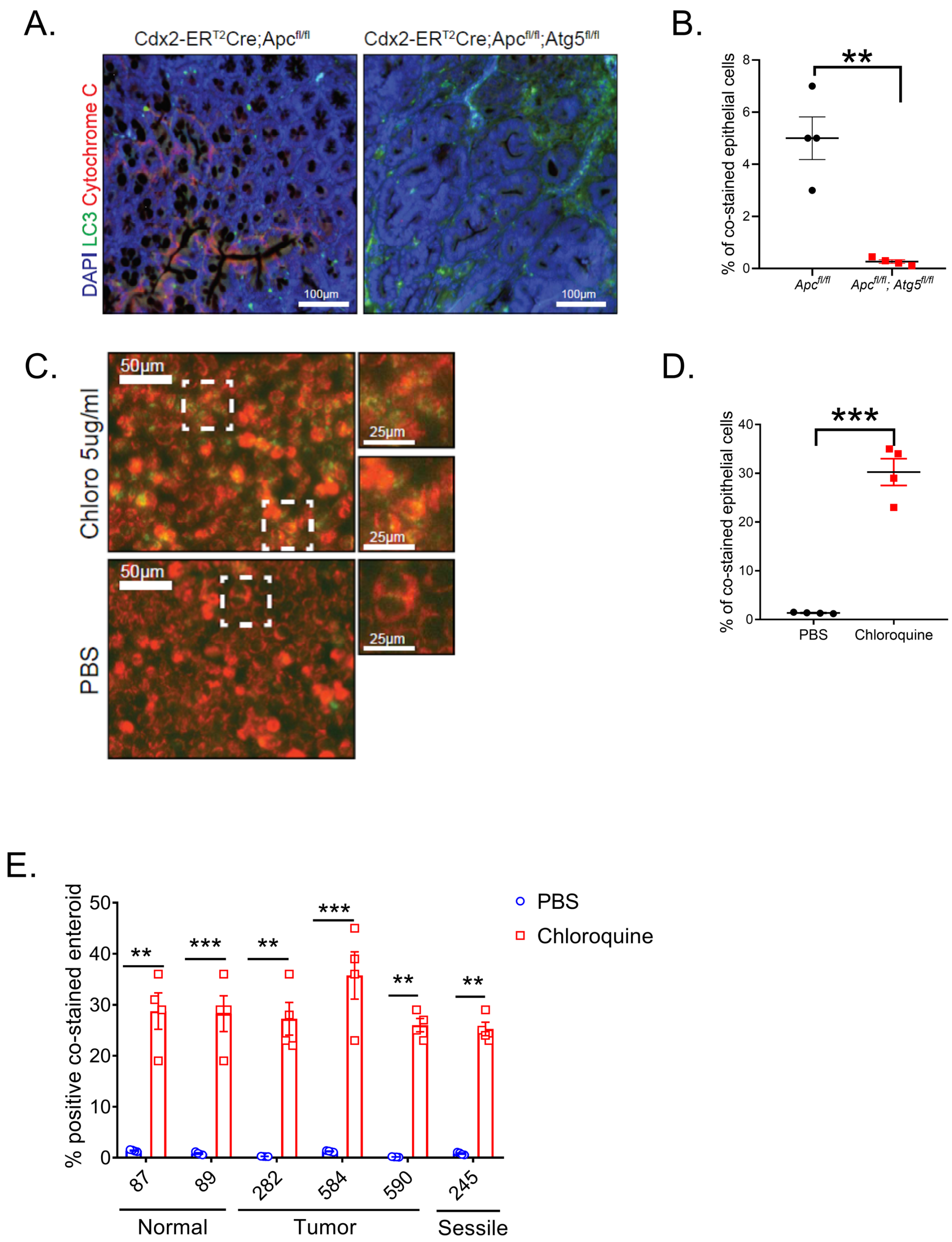

Figure S9 Mitophagy is altered in following ATG5 disruption or chloroquine treatment. A. Representative LC3 and cytochrome C co-staining and **B.** quantitation in *Cdx2-ER*^{T2}Cre;*Apc*$^{fl/fl}$ or *Cdx2-ER*^{T2}Cre;*Apc*$^{fl/fl}$;*Atg5*$^{fl/fl\,fl}$ mouse tumors. **C.** Co-staining and **D.** quantitation of mito- and lyso-tracker in HCT116 cells 24 hours of treatment of 5µg/mL of chloroquine. **E.** Quantitation of mito- and lyso-tracker in or normal and tumor enteroid lines following 24 hours of treatment of 75µg/mL of chloroquine. ** $p < 0.01$, *** $p < 0.001$ using unpaired t-test. Error bars represent SEM.

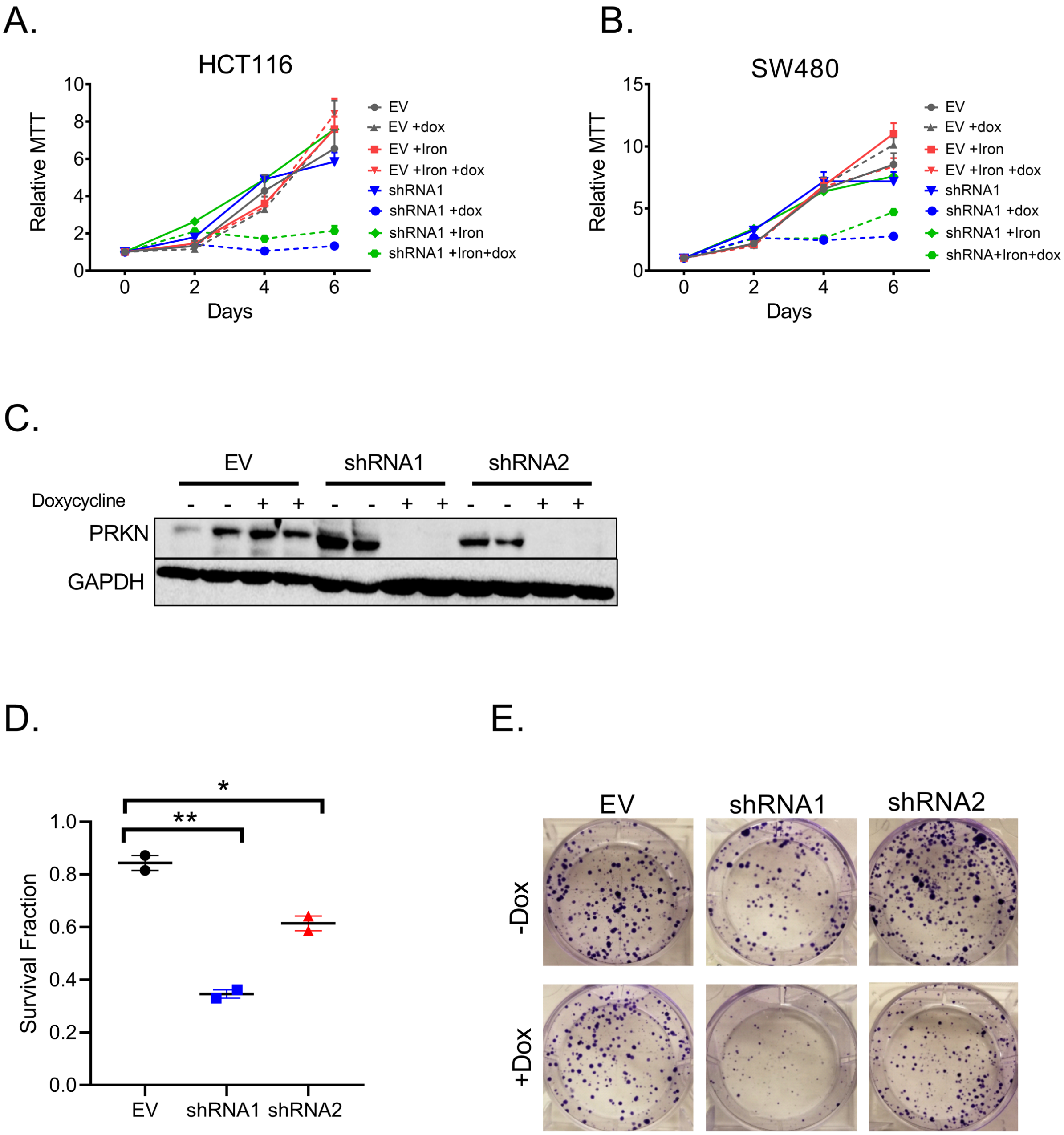

Figure S10 Mitophagy is necessary for CRC cell growth. MTT growth assay following co-treatment with doxycycline (Dox) and iron (100μM) in **A.** HCT116 and **B.** SW480 PINK1 KD cells. **C.** Western blot analysis of Parkin (PRKN) knockdown in HCT116. **D.** Quantification of clonogenics by blinded observers in doxycycline inducible shRNA for PRKN in HCT116, and **E.** representative image of clonogenic assay. * $p < 0.05$, ** $p < 0.01$ using unpaired t-test. Error bars represent SEM
