# Supplementary material for: Colorectal cancer cells utilize autophagy to maintain mitochondrial metabolism for cell proliferation under nutrient stress
Source: JCI Insight. 2021 Jun 17;6(14):e138835. doi: 10.1172/jci.insight.138835 (PMC8328084; doi:10.1172/jci.insight.138835)
Supplement: Supplemental Table 3 [file jciinsight-6-138835-s011.pdf]

Table S3

Gene	Primer
PRKN shRNA Fw	CCGGGCTTAGACTGTTTCCACTTATCTCGAGATAAGTG GAAACAGTCTAAGCTTTTT
PRKN shRNA 1 Rv	AATTAAAAAGCTTAGACTGTTTCCACTTATCTCGAGATA AGTGGAAACAGTCTAAGC
PRKN shRNA 2 Fw	CCGGGGAATGTAAAGAAGCGTACCACTCGAGTGGTAC GCTTCTTTACATTCCTTTTT
PRKN shRNA 2 Rv	AATTAAAAAGGAATGTAAAGAAGCGTACCACTCGAGTG GTACGCTTCTTTACATTCC
Lysozyme Fw	ATGGAATGGCTGGCTACTATGGAG
Lysozyme Rv	CTCACCACCCTCTTTGCACATTG
Cryptidins Fw	AGGAGCAGCCAGGAGAAG
Cryptidins Rv	ATGTTCAGCGACAGCAGAG
MMP7 Fw	CAGACTTACCTCGGATCGTAGTGG
MMP7 Rv	GTTCACTCCTGCGTCCTCACC
TFEB shRNA 1 Fw	CCGGGTCCGAGACCTATGGGAACAACTGCAGTT GTTCCCATAGGTCTCGGACTTTTT
TFEB shRNA 1 Rv	AATTAAAAAGTCCGAGACCTATGGGAACAACTGC AGTTGTTCCCATAGGTCTCGGAC
TFEB shRNA 2 Fw	CCGGCCTCTGTGGATTACATCCGGAGGATCTGC AGATCCTCCGGATGTAATCCACAGAGGTTTTT
TFEB shRNA 2 Rv	AATTAAAAACCTCTGTGGATTACATCCGGAGGATCTGC AGATCCTCCGGATGTAATCCACAGAGG

TFEB gRNA 1 Fw	CACCGATTGGGAGCACTGTTGCCAG
TFEB gRNA 1 Rv	AAACCTGGCAACAGTGCTCCCAATC
TFEB gRNA 2 Fw	CACCGGGACGACTCACTGCTACCGC
TFEB gRNA 2 Rv	AAACGCGGTAGCAGTGAGTCGTCCC
Cxcl2 Fw	TCCAGGTCAGTTAGCCTTGC
Cxcl2 Rv	CGGTCAAAAAGTTTGCCTTG
Il4 Fw	GGT CTC AAC CCC CAG CTA GT
Il4 Rv	GCC GAT GAT CTC TCT CAA GTG AT
Il6 Fw	ACCAGAGGAAATTTTCAATAGGC
Il6 Rv	TGATGCACTTGCAGAAAACA
Il1B Fw	AAGAGCTTCAGGCAGGCAGTATCA
Il1B Rv	TGCAGCTGTCTAGGAACGTCA
TNFa Fw	AGGGTCTGGGCCATAGAACT
TNFa Rv	CCACCACGCTCTTCTGTCTAC
Il10 Fw	AGACACCTTGGTCTTGGAGC
Il10 Rv	TTTGAATTCCCTGGGTGAGA
B220 Fw	TTCAGAAGCTGAACGTTGCACA
B220 Rv	TCTTCAGGAACCCCATGGTCTG
Il17 Fw	TGAGCTTCCCAGATCACAGA

Il17 Rv	TCCAGAAGGCCCTCAGACTA
Cdllb Fw	ATGGACGCTGATGGCAATACC
Cdllb Rv	TCCCCATTCACGTCTCCCA
Cd4 Fw	TCCTAGCTGTCACTCAAGGGA
CD4 Rv	TCAGAGAACTTCCAGGTGAAGA
Ym1 Fw	CACCATGGCCAAGCTCATTCTTGT
Ym1 Rv	TATTGGCCTGTCCTTAGCCCAACT
Cdllc Fw	CTGGATAGCCTTTCTTCTGCTG
Cdllc Rv	GCACACTGTGTCCGAACTCA
SAA4 Fw	CTCTGTTCTTTGTTCCTGGGAG
SAA4 Rv	CTAGGTTGTCCCGATAGGCTC
PINK1 shRNA 1 Fw	CCGGGAAATCTTCGGGCTTGTCAATCTCGAGATTGA CAAGCCCGAAGATTTCTTTTT
PINK1 shRNA 1 Rv	AATTAAAAAGAAATCTTCGGGCTTGTCAATCTCGAGA TTGACAAGCCCGAAGATTTC
PINK1 shRNA 2 Fw	CCGGGCCGCAAATGTGCTTCATCTACTCGAGTAGAT GAAGCACATTTGCGGCTTTTT
PINK1 shRNA 2 RV	AATTAAAAAGCCGCAAATGTGCTTCATCTACTCGAGT AGATGAAGCACATTTGCGGC
PINK1 Fw	CATGCCTACATTGCCCCAGA
PINK1 Rv	TGACTGCTCCATACTCCCCA
Tfeb Fw	CCAGAAGCGAGAGCTCACAGAT
Tfeb Rv	TGTGATTGTCTTTCTTCTGCCG

Vatp6v1d Fw	GAGCACAGACTGGTCGAAA
Vatp6v1d Rv	AGCTGTCAGTTCCTTCGTGG
Vatp6v1h Fw	ATGAGTACCGGTTTGCCTGG
Vatp6v1h Rv	GACTGAATGCCAGGAGCCAT
Vatp6v0e1 Fw	ATACCACGGCCTTACTGTGC
Vatp6v0e1 Rv	CAGAGGATTGAGCTGTGCCA
Becn1 Fw	CAGCCTCTGAAACTGGACACGA
Becn1 Rv	CTCTCCTGAGTTAGCCTCTTCC
SQSTM1 Fw	GAGGCACCCCGAAACATGG
SQSTM1 Rv	ACTTATAGCGAGTTCCCACCA
